# Unraveling the role of thermal fluctuations on the exciton structure of the cryptophyte PC612 and PC645 photosynthetic antenna complexes

**DOI:** 10.3389/fmolb.2023.1268278

**Published:** 2023-09-18

**Authors:** Beste Ozaydin, Carles Curutchet

**Affiliations:** ^1^ Departament de Farmàcia i Tecnologia Farmacèutica, i Fisicoquímica, Facultat de Farmàcia i Ciències de l’Alimentació, Universitat de Barcelona (UB), Barcelona, Spain; ^2^ Institut de Química Teòrica i Computacional (IQTCUB), Universitat de Barcelona (UB), Barcelona, Spain

**Keywords:** photosynthetic light harvesting, pigment-protein complexes, cryptophytes, multiscale QM/MM models, exciton states, molecular dynamics

## Abstract

Protein scaffolds play a crucial role in tuning the light harvesting properties of photosynthetic pigment-protein complexes, influencing pigment-protein and pigment-pigment excitonic interactions. Here, we investigate the influence of thermal dynamic effects on the protein tuning mechanisms of phycocyanin PC645 and PC612 antenna complexes of cryptophyte algae, featuring closed or open quaternary structures. We employ a dual molecular dynamics (MD) strategy that combines extensive classical MD simulations with multiple short Born-Oppenheimer quantum/molecular mechanical (QM/MM) simulations to accurately account for both static and dynamic disorder effects. Additionally, we compare the results with an alternative protocol based on multiple QM/MM geometry optimizations of the pigments. Subsequently, we employ polarizable QM/MM calculations using time-dependent density functional theory (TD-DFT) to compute the excited states, and we adopt the full cumulant expansion (FCE) formalism to describe the absorption and circular dichroism spectra. Our findings indicate that thermal effects have only minor impacts on the energy ladder in PC612, despite its remarkable flexibility owing to an open quaternary structure. In striking contrast, thermal effects significantly influence the properties of PC645 due to the absence of a hydrogen bond controlling the twist of ring D in PCB β82 bilins, as well as the larger impact of fluctuations on the excited states of MBV pigments, which possess a higher conjugation length compared to other bilin types. Overall, the dual MD protocol combined with the FCE formalism yields excellent spectral properties for PC612 and PC645, and the resultant excitonic Hamiltonians pave the way for future investigations concerning the implications of open and closed quaternary structures on phycocyanin light harvesting properties.

## 1 Introduction

Photosynthetic organisms rely on specialized pigment-protein antenna complexes to capture sunlight. The absorbed energy then undergoes electronic energy transfer (EET) steps, eventually reaching reaction centers with high quantum efficiencies (Croce and van Amerongen, 2014), despite the considerable diversity in antenna complexes in terms of size, structure and pigment composition (Curutchet and Mennucci, 2017; Mirkovic et al., 2017). Cryptophyte algae represent a unique group of photosynthetic organisms that originated from secondary endosymbiosis between an unknown eukaryotic host and a red algal symbiont. They replaced the primary red algal antenna, the phycobilisome, with a single antenna composed of two highly conserved β subunits originating from the phycobilisome and two more divergent α subunits, whose origin was largely unknown until recently, when they were linked to scaffolding proteins related to phycobilisome linker proteins (Greenwold et al., 1902; Rathbone et al., 2021). Remarkably, this algae display optimal photosynthetic activities at very low light conditions in marine and freshwater environments (Scholes et al., 2012). Despite this capacity, the pigments in these antenna complexes exhibit remarkable interpigment separations compared to the main antennae of other alga, bacteria or higher plants. Interestingly, over the last decade, a novel “open” quaternary structure of cryptohpyte antennae was discovered in various species of the *Hemiselmis* lineage. This structure involves the insertion of a single aspartic acid residue into the α subunit sequence, leading to a ∼73° rotation of the two αβ protomers, which translates into a significant decrease in the electronic interaction between central bilin pigments. Intriguingly, this structural change has also been linked to a decrease in observed quantum beatings on 2D electronic spectra, with potential implications in the involvement of vibronic coherence in the EET mechanisms (Harrop et al., 2014).

Beyond the switch between open and closed structures and variations in bilin composition, sequence variations in the α subunits provide further spectral tuning mechanisms that allow cryptophytes to adapt to different environments, as suggested by recent X-ray crystallography and multiscale simulations (Corbella et al., 2019; Michie et al., 2023). However, quantitative studies on the impact of α subunit sequences and quaternary structures displayed by different species on the excitonic structure and light harvesting mechanisms of cryptophyte antennae have been impeded by uncertainties in the precise location of the exciton states, especially regarding the lowest-energy ones in closed phycocyanins like PC630 and PC645 (Mirkovic et al., 2007; Lee et al., 2017).

Recently, we clarified the protonation properties of different bilin pigments present in cryptophyte complexes, including 15,16-dihydrobiliverdin (DBV), phycocyanobilin (PCB), mesobiliverdin (MBV) and phycoerythrobilin (PEB), and investigated the exciton structure in PC577, PC612, PC630, and PC645 by multiscale polarizable quantum/molecular mechanical (QM/MMPol) calculations (Corbella et al., 2018; Corbella et al., 2019). Underlying inaccuracies related to the choice of input geometries to be used in excited state calculations however severely hampers these protocols (Dreuw et al., 2010; Lee et al., 2017; Corbella et al., 2019; Cupellini et al., 2019). Inclusion of thermal effects can be achieved by performing classical molecular dynamics (MD) simulations of the protein complex, followed by post-processing to compute excite states with QM/MM models (Curutchet et al., 2013). Alternatively, QM/MM geometry optimization of the pigments can be done, which drastically improved results in PC577, PC612, PC630 and PC645 complexes, although at the price of neglecting thermal effects (Corbella et al., 2019). Indeed, important discrepancies with experiment remained, like unbalanced descriptions of the relative intensity of low and high-energy bands in PC577 and PC612, or a strong underestimation of the MBV bands in PC630 and PC645, which points to the impact of thermal effects and the limits of rationalizing spectral properties in terms of X-ray crystal structures (Curutchet et al., 2013; Corbella et al., 2019; Michie et al., 2023).

**FIGURE 1:**
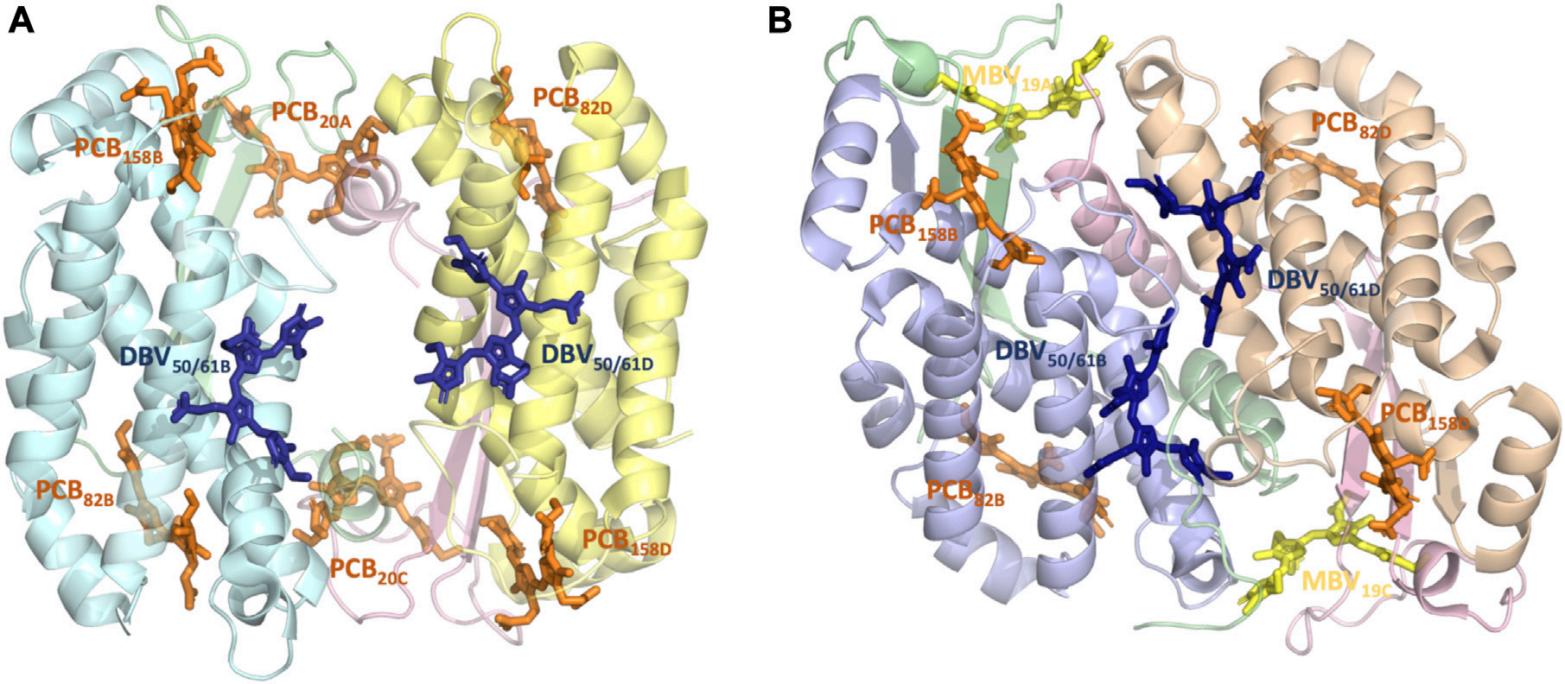
Structure and pigment composition of the phycocyanin cryptophyte antenna complexes **(A)** PC612 and **(B)** PC645.

In this study, we investigate the exciton properties of the *Hemiselmis virescens* PC612 and the *Chroomonas* PC645 antenna complexes shown in Figure 1 using a dual-MD multiscale protocol based on the combination of classical MD and Born-Oppenheimer (BOMD) simulations based on density functional theory (DFT). QM/MM MD simulations allow to describe with high accuracy the spectral density of electron-vibrational coupling in photosynthetic complexes and thus allow an accurate treatment of thermal effects (Rosnik and Curutchet, 2015; Blau et al., 2018; Loco et al., 2018; Maity et al., 2020; Maity et al., 2021a; Maity et al., 2021b; Sarngadharan et al., 2022). We also compare this approach with an alternative protocol based on multiple QM/MM geometry optimizations along the trajectories. Moreover, we adopt the full Cumulant Expansion (FCE) formalism to describe the absorption and circular dichroism spectra (Ma and Cao, 2015; Cupellini et al., 2020), which allows the inclusion of non-Markovian and non-secular effects that are neglected in modified Redfield theory. Overall, this new protocol yields absorption spectra with unprecedented accuracy for PC612 and PC645, providing an accurate excitonic Hamiltonian for future investigations addressing the implications of open and closed quaternary structures on phycocyanin light harvesting properties.

## 2 Materials and methods

### 2.1 Simulated systems, MD simulations and geometry optimizations

We based our simulations on high-quality crystal structures obtained for *Chroomonas* PC645 (PDB 4LMS, resolution 1.35 Å) and *Hemiselmis virescens* PC612 (PDB 4LM6, resolution 1.8 Å) reported by Curmi and co-workers, with the PC numbering indicating their maximum absorption wavelength (Harrop et al., 2014). The preparation of the systems and the details of the classical MD simulations are described in Corbella et al. (2018), Corbella et al. (2019). Both complexes have four polypeptide chains, but PC612 is organized as an (αβ)_2_ homodimer, while PC645 displays a (α_L_β)·(α_S_β) organization, where α_L_ and α_S_ indicate long and short α subunits, respectively (Michie et al., 2023). Additionally, PC612 features an open quaternary structure due to a ∼73° rotation of the αβ protomers, whereas PC645 exhibits a closed configuration more commonly found in cryptophyte antennae. In terms of pigment composition, PC612 contains six PCBs and two DBVs, while in PC645, two of the PCBs are replaced by MBVs. These pigments are covalently linked to a Cys, except for DBVs, which involve two covalent links with Cys residues. Therefore, pigments are denoted as PCB_82D_ or DBV_50/61B_, A/C and B/D indicating α and β chains, respectively, with numbering referring to the Cys attachment residues in that chain. All bilins were modelled with a fully protonated tetrapyrrole backbone and anionic propionic side chains, while all amino acids were considered in their standard protonation state, except His21 C in PC645.

We started from the 200 ns classical MD simulations reported in Ref. Corbella et al. (2019), performed using the ff14SB, (Maier et al., 2015), TIP3P (Jorgensen et al., 1983) and GAFF (Wang et al., 2004) force fields for the protein, water and bilin chromophores, respectively. Classical *NVT* simulations at 300 K were extended to a total of 1 μs, using a time step of 2 fs, the SHAKE algorithm, periodic boundary conditions, the particle-mesh Ewald approach and a non-bonded cutoff of 8 Å. From the last 500 ns, we extracted 34 frames at 14 ns intervals to be used as starting conditions for BOMD QM/MM simulations. Then, from each frame we run eight independent 1 ps BOMD simulations, where one of the bilin pigments was described at the B3LYP/6-31G level of theory, using a 0.5 fs time step. From the last 500 fs of BOMD trajectories, we extracted 25 frames at regular time intervals to be used as input geometries in QM/MMPol excited state calculations. This protocol is referred to as MD-BOMD. All runs were performed with the Amber20 (Case et al., 2020)—Gaussian 16 interface (Frisch et al., 2016).

On the other hand, QM/MM optimizations were independently performed for each chromophore geometry starting from the 34 frames extracted from the classical trajectories. We adopted the ONIOM substractive scheme with electrostatic embedding as implemented in Gaussian (Vreven et al., 2006). In each optimization, a given pigment and selected amino acids interacting with them were fully relaxed and described at the B3LYP/6-31G(d) level of theory, while the rest of the system was kept frozen and described with the classical force field adopted in the MD. For PCBs and DBVs in both complexes, we included the Asp or Glu side chains coordinating the central rings, whereas for MBVs in PC645 we included selected amino acids (Asn22 A for MBV19A and His21 C and Glu25 C for MBV19C). QM/MM boundaries were defined at the residue-residue and Cys-bilin bonds using the link atom scheme (Senn and Thiel, 2009). The optimized geometries were used as inputs in subsequent QM/MMPol excited state calculations. This protocol is denoted as MD-OPT.

### 2.2 QM/MMPol calculations

Excited state calculations were performed using the QM/MMPol model (Curutchet et al., 2009) in its time dependent density functional theory (TD-DFT) linear response formulation. In the MMPol model, the environment is described using a polarizable force field based on the induced point dipole model, which implies assignment of point charges and isotropic polarizabilities to the environment atoms, and accounts full mutual polarization effects among the QM and MM regions. QM/MMPol calculations on structures extracted from BOMD trajectories and ONIOM optimizations were performed at the TD-CAM-B3LYP/6-31G(d) level of theory (Yanai et al., 2004). Previous work showed that different global and range-separated hybrid functionals with different degrees of exact exchange (M06, M06-2X and ωB97XD) led to similar results in phycobiliproteins (Corbella et al., 2019). The MMPol region was described using the Amber pol12 AL parameters (Wang et al., 2011a; Wang et al., 2011b), and QM/MM boundaries in the bilin-Cys bonds were treated using the link atom scheme (Senn and Thiel, 2009). Atomic charges for water and bilins, consistent with the Amber pol12 AL polarizabilities, were derived as described in Ref. Corbella et al. (2019). Explicit polarization was limited to residues within a cutoff radius of 12 Å from the QM heavy atoms (MMPol region), whereas residues up to a 35 Å were also included but adopting the additive force field (MM region). MMPol calculations were performed using a locally modified development version of the Gaussian package (Frisch et al., 2010).

### 2.3 Modelling of steady-state spectra

Simulations of absorption (OD), circular dichroism (CD) and fluorescence (FLU) spectra were performed using the EXAT (Jurinovich et al., 2018) and the FCE codes (Arpin et al., 2015; Cupellini et al., 2020). EXAT simulations were based on modified Redfield theory, whereas FCE ones include non-Markovian and non-secular effects as described in Refs. Ma and Cao (2015), Cupellini et al. (2020). We used the following excitonic Hamiltonian describing the multichromophoric system:

$$\hat{H}=\sum_{n=1}^{N}\varepsilon_{n}\left|n\rangle \langle n\right|+\sum_{n\neq m}^{N}V_{nm}\left|n\rangle \langle m\right|$$ (1)

Where N is the number of interacting chromophores, $\varepsilon_{n}$ is the excitation energy of the *n*th chromophore and $V_{nm}$ is the coupling between the *n*th and *m*th chromophores. Exciton states $\left|k\rangle \right.$ were obtained from diagonalization of the excitonic Hamiltonian:

$$\hat{H}=\sum_{k=1}^{N}\varepsilon_{k}\left|k\rangle \langle k\right|;\;|k\rangle =\sum_{n=1}^{N}C_{n}^{k}|n\rangle$$ (2)

where $\varepsilon_{k}$ is the energy of the *k*th exciton state and $C_{n}^{k}$ describes the participation of the *n*th chromophore to the *k*th exciton state.

We used the site energies averaged for each pigment over the 850 or 34 frames calculated using the dual quantum/classical MD (MD-BOMD set) or the ONIOM optimization protocols (MD-OPT set), respectively, and results were also compared to the energies estimated previously based on classical MD (MD set) or a single optimization performed on the crystal structure (Crystal-OPT set) in Ref. Corbella et al. (2019). Electronic couplings, transition dipole moments and pigment center coordinates were taken from previous QM/MMPol/ddCOSMO calculations based on the Crystal-OPT protocol and include dielectric screening effects exerted by the surrounding environment. Beyond the DBV central pair in PC645, the pigment pairs in these complexes are not strongly coupled, and the adoption of the MD and Crystal-OPT sets of couplings and dipoles led to negligible differences in predicted spectra. Even the complete neglect of couplings leads to small changes, as shown in Supplementary Figure S13. For this reason, we do not recompute the couplings along the many MD-OPT optimizations. We also adopt the transition dipole moments from the Crystal-OPT set for consistency, but these values are very similar to those obtained by the MD-OPT and MD-BOMD protocols, as shown in Supplementary Table S4, so this choice leads to negligible changes in predicted spectra. On the other hand, BOMD simulations are performed for single pigments, so recalculation of electronic couplings on those geometries would lead to an unbalanced description of the pigment pair.

Electronic-vibrational coupling was accounted for using spectral densities for the intramolecular part calculated using the Vertical Gradient approach, while the continuous part due to slow environmental motions was added *a posteriori* using an overdamped Brownian oscillator with parameters adjusted to reproduce the Stokes shift, as described in Ref. Corbella et al. (2019). Finally, Static disorder was modeled by averaging realizations of the spectra over a random distribution of site energies characterized by a given standard deviation σ, which was adjusted to reproduce the broadening of the experimental emission lineshapes for each complex or estimated from the variation of site energies sampled from the MD-Opt set.

## 3 Results and discussion

### 3.1 Structural flexibility of open and closed phycocyanins

Recent studies have suggested that the sequence diversity of α subunits plays a significant role in determining the chromophore conformations observed in X-ray crystal structures and, consequently, the spectral properties of cryptophyte complexes (Michie et al., 2023). To investigate the impact of thermal effects and quaternary structure on the light-harvesting properties of PC612 and PC645 complexes, we conducted extensive classical and QM/MM MD simulations. These simulations allowed us to analyze and compare the structural flexibility of the open PC612 and closed PC645 complexes.

**FIGURE 2:**
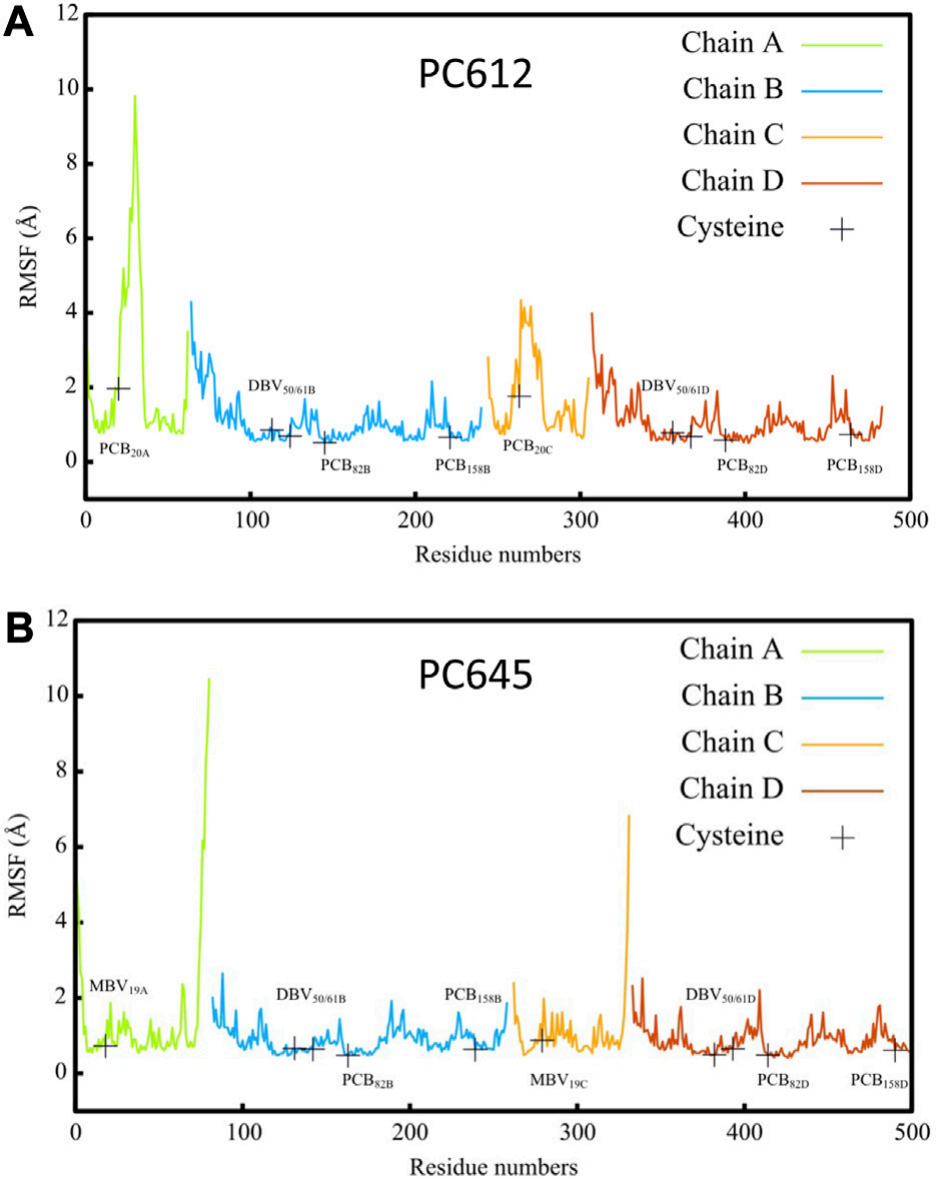
Root mean square fluctuation (RMSF) of atomic positions calculated along classical MD trajectories for α A/C and β B/D subunits of antenna complexes **(A)** PC612 and **(B)** PC645.

Figure 2 shows the root-mean-square fluctuations (RMSF) calculated along the classical MD trajectories for the α and β subunits of PC612 and PC645. The open arrangement of the two αβ protomers in PC612 leads to higher flexibility in all subunits compared to PC645, which has a closed structure. Notably, in the β subunits B and D of PC612, there is increased flexibility in the N-terminal regions near PCB_20A_ and PCB_20C_, where they interact with Tyr18 B and Tyr18 D through their propionic groups. In contrast, the C-terminal regions of the α subunits A and C in PC612 appear more rigid, despite the open quaternary arrangement. However, regions containing residues ∼20–40 in chains A and C, starting at the anchoring points of PCB_20A_ and PCB_20C_ (Cys20 A and Cys20 C), show considerably more flexibility in PC612 compared to PC645. In PC612, these regions form loops that mostly interact with PCB_20A_ and PCB_20C_, partially shielding them from the solvent. This disposition is similar to that observed in PC645, which has the closed structure. However, in PC612, PCB_20A_ and PCB_20C_ are considerably more exposed to the solvent than MBV_19A_ and MBV_19C_ in PC645. Consequently, increased flexibility of PCBs in the open PC612 complex, likely due to higher solvent exposure, leads to more flexible protein loops. The RMSF values corresponding to the pigments in PC612 and PC645 (Supplementary Table S1 of the Supplementary Material), confirm that increased fluctuations in the open protein PC612 result in more flexible pigments, particularly PCB_20_ bilins in α subunits, which display significantly larger fluctuations than the MBVs in PC645.

### 3.2 Spectral tuning of bilins by the protein scaffold

**FIGURE 3:**
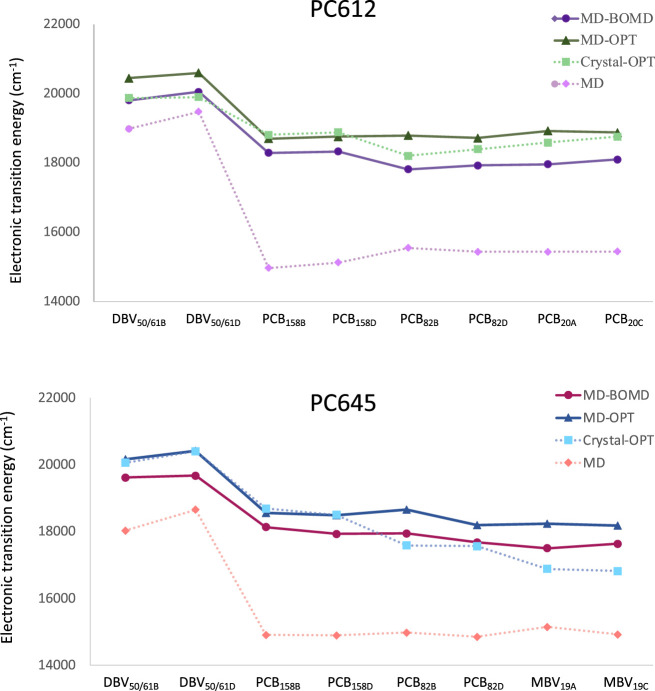
Electronic transition energies computed at the QM/MMPol TD-CAM-B3LYP/6-31G(d) level of theory for cryptophyte complexes using geometries obtained using the MD-BOMD and MD-OPT protocols compared to previous results based on classical MD or a single optimization of the bilins in the crystal structure (Corbella et al., 2019): **(A)** PC612 and **(B)** PC645.

Next, we investigated how thermal fluctuations and protein flexibility influence the electronic excited states of the bilin pigments. Figure 3 presents the site energies (uncoupled transition energies) of the pigments computed from QM/MMPol TD-CAM-B3LYP/6-31G(d) calculations for PC612 and PC645. We compare the results obtained from classical MD geometries (MD set) or a single optimization of the bilins in the protein scaffold based on the crystal structure (Crystal-OPT set) (Corbella et al., 2019) with those obtained from multiple geometry optimizations of the pigments performed along classical MD trajectories (MD-OPT set) and short BOMD DFT-based simulations (MD-BOMD set). In addition, in the Supplementary Tables S2, S4 and Supplementary Figures S4–S11 we provide the averaged values and the complete distributions of site energies and transition dipole moments obtained from both sets.

In PC612, both the MD-BOMD and MD-OPT methodologies yielded similar site energies, closely matching those obtained using the Crystal-OPT strategy. This indicates that the energy ladder of PC612 is well explained by the crystal structure and the pigment-protein interactions, which constrain the bilin pigments to specific conformations. On the other hand, in PC645, the MD-BOMD and MD-OPT protocols significantly modify the picture provided by previous studies. While the energies of DBV and PCB_158_ pairs agree with previous values based on the crystal, the energetic position of PCB_82_ and MBV_19_ pairs is significantly modified, suggesting a crucial role of thermal effects. In terms of transition dipole moments, however, both MD-OPT and MD-BOMD sets provide results very similar to the Crystal-OPT ones on both complexes, as shown in Supplementary Table S4. Thus, despite the considerable distributions of dipoles obtained, which are comparable to those observed in site energies, as shown in Supplementary Figures S4–S11, the averaged values turn to be very close in all cases to those based on the crystal structure.

To explain the impact of thermal effects in the site energies of the PCB_82_ and MBV_19_ pairs in PC645, it is interesting to note that comparison of the X-ray crystal structures of PC645 and a similar complex, PC630, showed that in the latter there is a hydrogen bond between ring D in PCB β82 chromophores and Gln6 in the α_L_ and α_S_ subunits, whereas in PC645 Gln6 is replaced by Leu5, and the lack of this hydrogen bond is the responsible for the red shift of the absorption spectra of PC645 compared to PC630 (Michie et al., 2023). Here, we show that beyond this analysis, thermal fluctuations of the protein environment are key to obtain a balanced description of β82 PCB chromophores and to explain the intriguing question of why α19 MBVs in PC645 have site energies very close to the PCBs, despite their larger degree of conjugation. Indeed, bilin excitation energies are strongly controlled by the conformation of the tetrapyrrole backbone, and hydrogen bonds and steric clashes with the protein scaffold can twist the bonds connecting pyrrole rings, thus decreasing the conjugation of the π system, which increases the transition energies of the π→π* states.

**FIGURE 4:**
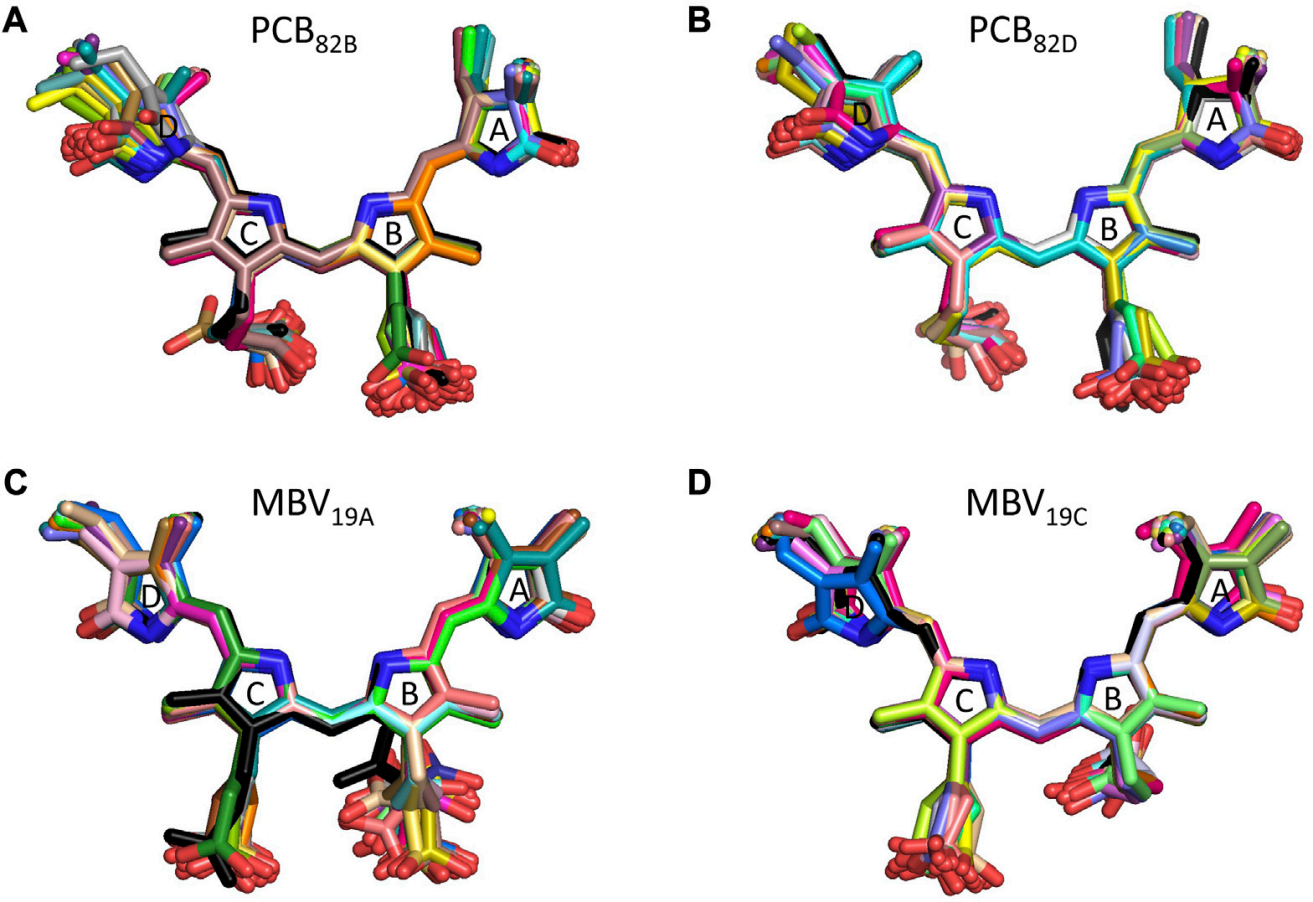
Alignment of PC645 pigment structures in the crystal structure (black) and optimized along the MD simulation (colors): **(A)** PCB_82B_, **(B)** PCB_82D_, **(C)** MBV_19A_ and **(D)** MBV_19C_.

**FIGURE 5:**
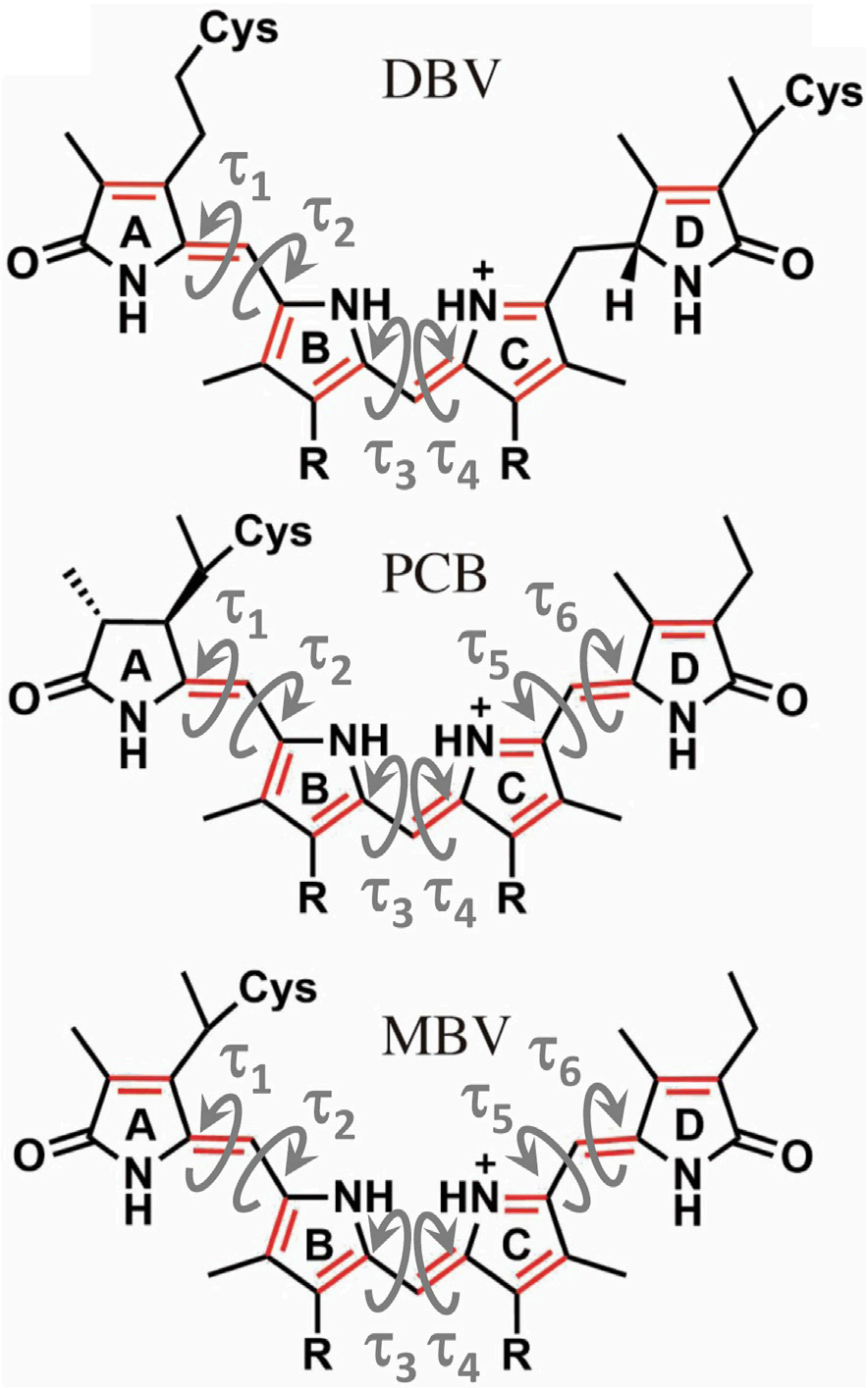
Structure of the linear tetrapyrrole arrangement of DBV, PCB and MBV bilin pigments indicating the key torsions that define the conformation of the conjugated region that characterizes the excited state.

To understand the large impact of thermal effects on β82 PCB and α19 MBVs energy levels in PC645, in Figure 4 we show an alignment of their structures as found in the crystal structure or optimized along the MD simulation following the MD-OPT protocol. In addition, in Table 1 and Table 2 we report the dihedral angles of the bonds connecting the bilin pyrrole rings (defined in Figure 5) obtained in the MD-BOMD calculations of PC612 and PC645 compared to the crystal references, as has been done in previous analyses of biliproteins either based on simulations or crystal structures (Curutchet et al., 2013; Michie et al., 2023).

**TABLE 1:** Averaged dihedral angles and their standard deviations computed from DFT-based BOMD simulations characterizing the twists among pyrrole units in the pigments of PC612. For comparison, the values corresponding to the crystal structure are provided in parentheses.

	τ_1_	τ_2_	τ_3_	τ_4_	τ_5_	τ_6_
DBV_50/61B_	183 ± 9 (182)	32 ± 11 (20)	172 ± 10 (190)	182 ± 11 (168)	—	—
DBV_50/61D_	178 ± 9 (178)	34 ± 11 (23)	181 ± 9 (186)	179 ± 9 (171)	—	—
PCB_158B_	178 ± 6 (171)	−36 ± 14 (−20)	180 ± 7 (176)	181 ± 6 (178)	−32 ± 12 (−40)	178 ± 5 (183)
PCB_158D_	177 ± 7 (171)	−39 ± 13 (−34)	184 ± 10 (179)	178 ± 8 (177)	−40 ± 11 (−43)	173 ± 9 (181)
PCB_82B_	186 ± 7 (186)	−20 ± 11 (−24)	179 ± 9 (173)	187 ± 8 (189)	−33 ± 10 (−37)	170 ± 9 (175)
PCB_82D_	186 ± 7 (189)	−18 ± 12 (−25)	177 ± 8 (174)	188 ± 9 (188)	−33 ± 11 (−38)	176 ± 8 (186)
PCB_20A_	184 ± 6 (173)	11 ± 19 (−36)	184 ± 8 (190)	190 ± 10 (178)	−41 ± 11 (−33)	180 ± 9 (181)
PCB_20C_	175 ± 8 (174)	−34 ± 15 (−37)	184 ± 10 (187)	179 ± 9 (179)	−40 ± 10 (−33)	176 ± 8 (186)

**TABLE 2:** Averaged dihedral angles and their standard deviations computed from DFT-based BOMD simulations characterizing the twists among pyrrole units in the pigments of PC645. For comparison, the values corresponding to the crystal structure are provided in parentheses.

	τ_1_	τ_2_	τ_3_	τ_4_	τ_5_	τ_6_
DBV_50/61B_	190 ± 8 (195)	27 ± 11 (20)	190 ± 9 (187)	168 ± 8 (174)	—	—
DBV_50/61D_	193 ± 8 (194)	33 ± 11 (34)	176 ± 9 (185)	187 ± 9 (179)	—	—
PCB_158B_	176 ± 8 (174)	−37 ± 12 (−34)	182 ± 9 (181)	182 ± 8 (179)	−34 ± 11 (−27)	168 ± 9 (168)
PCB_158D_	171 ± 7 (171)	−36 ± 10 (−35)	187 ± 8 (189)	179 ± 8 (172)	−34 ± 11 (−25)	168 ± 9 (168)
PCB_82B_	183 ± 8 (187)	−24 ± 11 (−29)	179 ± 8 (181)	185 ± 9 (188)	27 ± 19 (−12)	189 ± 9 (201)
PCB_82D_	182 ± 8 (191)	−23 ± 11 (−28)	179 ± 8 (179)	186 ± 8 (182)	11 ± 16 (−6)	188 ± 9 (203)
MBV_19A_	182 ± 8 (184)	31 ± 11 (29)	181 ± 9 (185)	181 ± 8 (183)	−39 ± 10 (−48)	182 ± 3 (187)
MBV_19C_	186 ± 8 (187)	29 ± 11 (23)	178 ± 9 (179)	184 ± 9 (183)	−43 ± 11 (−45)	175 ± 8 (176)

**FIGURE 6:**
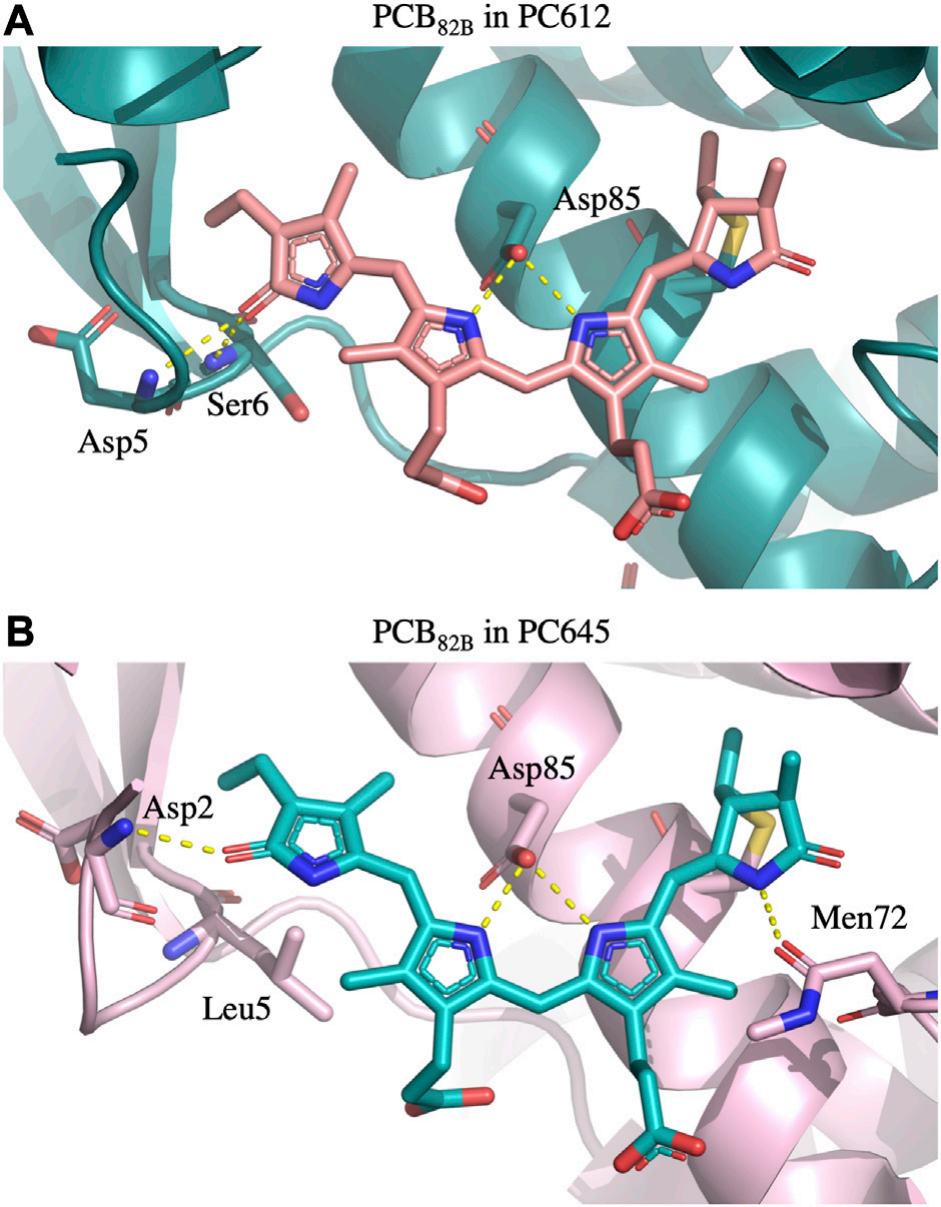
Hydrogen bonds established by PCB β82 bilins with the protein scaffold in **(A)** PC612 and **(B)** PC645. In PC612, the carbonyl group of ring D interacts with Asp5 and Ser6, stabilizing a considerable twist between rings C and D. In contrast, in PC645 the presence of Leu5 allows significantly larger fluctuations, which are not completely stabilized by the interaction with Asp2.

Overall, Figure 4 indicates that there are no large conformational changes of the β82 PCB and α19 MBVs bilins in PC645 compared to the crystal reference, especially regarding the central B and C pyrrole rings. However, rings A and D and the propionic side chains display remarkable fluctuations compared to the crystal reference. Remarkable fluctuations are especially observed for ring D (shown at the left) in PCB_82_ bilins, which is confirmed by a standard deviation of ∼16–19 degrees in the torsion τ_5_ reported in Table 2, which is almost twice the value observed in other bilins. As previously discussed, in PC645 the nitrogen atom of ring D in PCB β82 bilins interacts with Leu5, missing the two hydrogen bonds with Ser6 and Asp5 found in PC612 shown in Figure 6. In PC645 there is indeed a hydrogen bond with Asp2, but its arrangement does not prevent considerable twists among the planes of C and D rings. Thus, in PC645 the lack of a hydrogen bond controlling the twist of ring D leads to larger fluctuations in PCB_82_ bilins that break the conjugation of the tetrapyrrole backbone, leading to a significant increase in the electronic transition energies of the π→π* states, as shown in Figure 3. In MBVs, even stronger thermal effects are observed than in PCB_82_s. In this case, however, the fluctuations of rings A and D are comparable, and the strong impact of thermal effects seems to be linked to the fact that MBVs have a higher conjugation length that extends to ring A, so thermal fluctuations in the twist of that ring necessarily lead to a stronger impact than that found for DBVs or PCBs. Indeed, beyond the case of PCB_82_s in PC645 discussed above, Table 1 and Table 2 show similar standard deviations for the torsions of MBVs and the other bilins in PC612 and PC645, with somewhat larger values for PCB_20_ and PCB_158_ pigments in PC612, which can be explained by the overall larger flexibility of the open structure of this complex.

**FIGURE 7:**
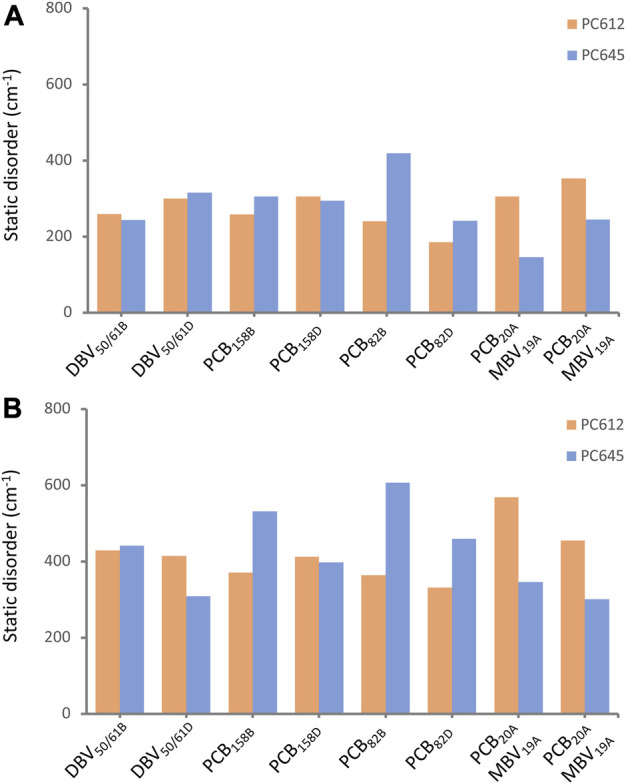
Static disorder (cm^−1^) estimated from **(A)** MD-OPT and **(B)** MD-BOMD averaged transition energies computed along the classical MD trajectories of PC612 and PC645.

In addition to intramolecular fluctuations of the pigments, we analyzed how the quaternary structure of PC612 and PC645 and collective protein motions impact static disorder. Figure 7 shows an estimate of static disorder obtained as the standard deviation of site energies computed along the many MD-OPT optimized geometries. We also provide a similar analysis based on the energies averaged over different BOMD QM/MM simulations. In the latter, short BOMD runs necessarily translate into overestimated disorder values, but the comparison between both protocols shows similar trends that suggests they both capture similar physics in the complexes. Indeed, both analyses show a similar disorder for DBV_50/61_ and PCB_158_ pairs in the two proteins, but opposite trends for PCB_82_ and the PCB/MBV α19/20 pairs. The increased flexibility and solvent exposure of PCB_20_ in the open structure of PC612 compared to MBV_19_ in PC645 seems to be the reason for the increased disorder. In contrast, in PC645, despite the closed quaternary structure leading to an overall more rigid protein, PCB_82_ pigments consistently display larger disorder compared to PC612. As discussed previously, PCB_82_ bilins in PC645 lack a key hydrogen bond with α_L_ and α_S_ subunits that lead to larger fluctuations in the twist of the bonds linking rings C and D, which explains such increased disorder. We note that our best estimates for disorder, shown in Figure 7, are larger than the value of 100 cm^−1^ we derived previously from a fit between experiment and simulations of absorption and emission spectra (Corbella et al., 2019). In the next section we discuss the reliability of the site energies and the disorder values in describing the optical spectra of the complexes.

### 3.3 Absorption and circular dichroism spectra

**FIGURE 8:**
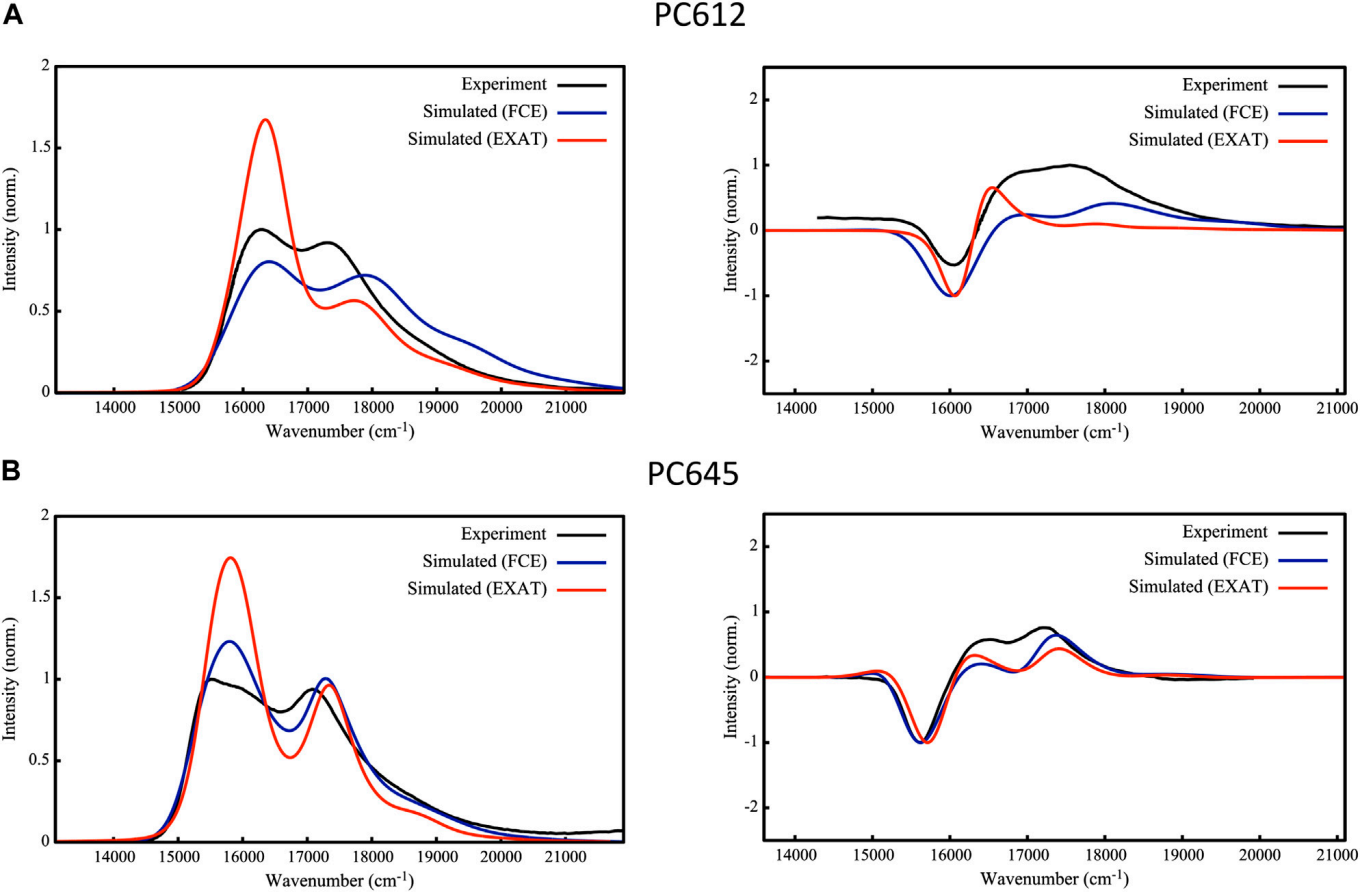
Experimental (Mirkovic et al., 2007; Harrop et al., 2014; Arpin et al., 2015) and simulated absorption (left) and circular dichroism (right) spectra of **(A)** PC612 and **(B)** PC645 cryptophyte antenna complexes. Simulations are based on energies and couplings computed from QM/MMPol TD-CAM-B3LYP/6-31G(d) calculations performed on MD-BOMD geometries using the EXAT or FCE codes to calculate the spectra. We applied the following shifts to simulated spectra to fit the experimental bands: 1,600 cm^−1^ (EXAT PC612), 1,000 cm^−1^ (FCE PC612), 1,700 cm^−1^ (EXAT PC645) and 1,480 cm^−1^ (FCE PC645). Static disorder was modeled by applying a common σ = 100 cm^−1^.

To validate the excitonic Hamiltonian derived for PC612 and PC645 based on the MD-BOMD and MD-OPT site energies, we simulated OD and CD spectra of the complexes and compared them to experiment. In Figure 8 we report our most accurate estimates, based on the dual classical/quantum MD strategy, the MD-BOMD protocol, which, in contrast to previous studies, allows to accurately account for thermal effects by avoiding the geometry mismatch problem related to the use of MD trajectories based on classical force fields (Corbella et al., 2019). In addition, here we adopt a more rigorous theory to simulate excitonic spectra, the full Cumulant Expansion (FCE) formalism (Ma and Cao, 2015; Cupellini et al., 2020), which allows including non-Markovian and non-secular effects neglected in modified Redfield theory. To gauge the impact of these effects, we also report spectra based on modified Redfield theory obtained using the EXAT (Jurinovich et al., 2018) code.

The performance of the MD-BOMD set of site energies, combined with the FCE formalism, in describing the OD spectrum is astonishing. For both complexes, we obtain an excellent agreement with experiment, and our simulations reproduce with unprecedented accuracy the position of the two main peaks, their relative intensities, and the overall broadening in the spectra. This is drastic improvement over previous simulations reported on these systems (Corbella et al., 2019), in which the absence of thermal effects led, for example, to a strong underestimation of MBV energies in PC645, too large differences among PCB_158_ and PCB_82_ bands, and overestimated DBV energies. For PC612, previous reports were more accurate, but both peaks were too close leading to an overall large band. In terms of CD, again the MD-BOMD simulations led to the correct positions of the three bands observed experimentally, which are instead obscured in the OD spectra dominated by two bands. Interestingly, the MD-OPT protocol also leads to accurate spectra very similar to that obtained using the MD-BOMD benchmark protocol, as shown in Supplementary Figure S12 of the Supporting Information. Because the multiple geometry optimizations in this protocol do not allow to fully account for thermal effects associated to intramolecular vibrations of the pigments, we conclude that accounting for the collection of protein conformations is the key aspect to be accounted for, which is not included in analysis limited to the crystal structure.

As we extensively discuss in the previous section, thermal effects seem to be key to correctly describe the energies of PCB_82_ and MBV bilins in PC645. Accounting for non-Markovian and non-secular effects through the FCE formalism however seems to be also important to correctly describe the relative intensities of the two main absorption peaks in OD spectra, as shown in Figure 8, where EXAT simulations overestimate the intensity of the low energy band. A similar improvement is also obtained in the relative intensities of CD bands, especially in PC612. In addition, whereas for PC612 FCE and EXAT lead to similar OD lineshapes in the blue edge of the spectrum, for PC645 the FCE formalism provides a more accurate lineshape. This can be ascribed to a better description of the coupling between vibrations and the exciton states delocalized on the central DBV_50/61B_—DBV_50/61D_ dimer in PC645, characterized by a strong electronic coupling of 238 cm^−1^ (Corbella et al., 2019). In the open quaternary structure of PC612 this coupling is attenuated to a 16 cm^−1^, leading to mostly localized states. This is clearly visible in Supplementary Figure S13, where we compare the OD spectra to simulations in which all couplings are zeroed. Whereas neglecting excitonic couplings do not change much PC612 spectra, characterized by localized states, the impact on PC645 is large, namely, in the high-energy band characterized by the DBV_50/61B_—DBV5_0/61D_ strongly coupled dimer.

Finally, we recall that the theoretical estimates of static disorder shown in Figure 7 are larger than the value of 100 cm^−1^ used in the simulations reported in Figure 8, previously fitted by comparison to absorption and emission experimental spectra (Corbella et al., 2019). In Supplementary Figures S14–S16 in the Supplementary Material, we report OD, CD and FLU spectra obtained adopting our theoretical estimates of disorder based on MD-OPT simulations. In all cases the OD spectra displays minor changes. For CD and FLU, in PC612 we also obtain a reasonable broadening. However, the CD and FLU spectra of PC645 is slightly too broad, suggesting our methodology still has limitations to estimate this important parameter, probably because simulations are based on MDs spanning a limited μs timescale.

## 4 Conclusion

In this study, we investigated the role of thermal effects on the spectral tuning mechanisms of PC645 and PC612 antenna complexes from cryptophyte algae. To accurately account for thermal effects arising from protein (slow) and pigment (fast) structural fluctuations, we have applied a dual MD strategy to determine the site energies that combines classical MD simulations to sample the conformational space of the complex on the μs timescale, with short Born-Oppenheimer QM/MM simulations on the ps timescale, which allowed us to accurately describe the impact of pigment’s vibrations on the relevant excited states. We have also investigated the performance of an alternative method, based on multiple QM/MM geometry optimizations of the pigments performed along the classical MD trajectories. The resulting structures of both protocols were then used to evaluate the excited states of the pigments using polarizable QM/MM TD-DFT calculations, and the excitonic Hamiltonians were used in subsequent spectral simulations of absorption and circular dichroism based on the FCE formalism. Interestingly, we found that thermal effects only lead to small corrections to the energy ladder in PC612, despite the remarkable flexibility of the PC612 complex, characterized by an open quaternary structure. In contrast, thermal effects are key to describe the properties of the closed PC645 complex due to the lack of a hydrogen bond controlling the twist of ring D in PCB β82 bilins, as well as the larger impact of fluctuations on the excited states of MBV pigments, characterized by a higher conjugation length compared to PCB and DBV bilins found in PC612. Overall, the dual MD protocol combined with FCE spectral simulations led to excellent spectral properties for PC612 and PC645 of unprecedented accuracy. The resulting excitonic Hamiltonian paves the way for future investigations addressing the implications of open and closed quaternary structures on phycocyanin light harvesting properties, with particular interest in addressing the role of vibronic coherences in their excitation transfer mechanisms.

## Data Availability

The original contributions presented in the study are included in the article/Supplementary Material, further inquiries can be directed to the corresponding author.
