# Supplementary material for: Unraveling the role of thermal fluctuations on the exciton structure of the cryptophyte PC612 and PC645 photosynthetic antenna complexes
Source: Front Mol Biosci. 2023 Sep 18;10:1268278. doi: 10.3389/fmolb.2023.1268278 (PMC10544999; doi:10.3389/fmolb.2023.1268278)
Supplement: Supplementary file 1 [file DataSheet1.pdf]

Supporting Information for

Unraveling the role of thermal fluctuations on the exciton structure of the cryptophyte PC612 and PC645 photosynthetic antenna complexes

Beste Ozaydin[1,2] and Carles Curutchet[1,2]*

1 Supplementary Figures and Tables

1.1 Supplementary Tables

Supplementary Table S1. Root-mean square fluctuations (RMSF) calculated along classical MD trajectories for the pigments in PC612 and PC645.

PC612	RMSF (Å)	PC645	RMSF (Å)
$DBV_{50/61B}$	0.96	$DBV_{50/61B}$	0.76
$DBV_{50/61D}$	0.94	$DBV_{50/61D}$	0.84
PCB_{158B}	0.93	PCB_{158B}	0.87
PCB_{158D}	1.03	PCB_{158D}	0.85
PCB_{82B}	0.78	PCB_{82B}	0.80
PCB_{82D}	0.91	PCB_{82D}	0.78
PCB_{20A}	2.02	MBV_{19A}	0.81
PCB_{20C}	1.30	MBV_{19C}	0.79

Supplementary Table S2. Average MD-OPT and MD-BOMD site energies (cm^{-1}) computed along the classical MD trajectories of PC612 and PC645.

PC612	MD-BOMD	MD-OPT	PC645	MD-BOMD	MD-OPT
$DBV_{50/61B}$	19812	20452	$DBV_{50/61B}$	19614	20166
$DBV_{50/61D}$	20050	20598	$DBV_{50/61D}$	19674	20407
PCB_{158B}	18290	18702	PCB_{158B}	18134	18555
PCB_{158D}	18331	18760	PCB_{158D}	17924	18484
PCB_{82B}	17817	18794	PCB_{82B}	17942	18657
PCB_{82D}	17932	18719	PCB_{82D}	17675	18196
PCB_{20A}	17964	18926	MBV_{19A}	17497	18228
PCB_{20C}	18102	18876	MBV_{19C}	17629	18175

Supplementary Table S3. Standard deviation (cm^{-1}) of MD-OPT and MD-BOMD site energies computed along the classical MD trajectories of PC612 and PC645.

PC612	MD-BOMD	MD-OPT	PC645	MD-BOMD	MD-OPT
$DBV_{50/61B}$	429	259	$DBV_{50/61B}$	441	243
$DBV_{50/61D}$	414	299	$DBV_{50/61D}$	308	315
PCB_{158B}	370	258	PCB_{158B}	531	305
PCB_{158D}	412	305	PCB_{158D}	397	294
PCB_{82B}	364	240	PCB_{82B}	606	419
PCB_{82D}	331	185	PCB_{82D}	459	241
PCB_{20A}	568	305	MBV_{19A}	346	146
PCB_{20C}	455	352	MBV_{19C}	301	244

Supplementary Table S4. Average MD-OPT and MD-BOMD electronic transition dipole moments (Debye) computed along the classical MD trajectories of PC612 and PC645.

PC612	MD-BOMD	MD-OPT	PC645	MD-BOMD	MD-OPT
$DBV_{50/61B}$	12.9	13.1	$DBV_{50/61B}$	13.1	13.2
$DBV_{50/61D}$	12.6	12.8	$DBV_{50/61D}$	13.0	13.1
PCB_{158B}	13.5	14.0	PCB_{158B}	14.1	14.3
PCB_{158D}	13.8	14.0	PCB_{158D}	14.2	14.4
PCB_{82B}	14.4	14.4	PCB_{82B}	14.2	14.3
PCB_{82D}	14.4	14.4	PCB_{82D}	14.5	14.7
PCB_{20A}	13.6	13.6	MBV_{19A}	14.6	14.5
PCB_{20C}	14.0	14.2	MBV_{19C}	14.3	14.5

1.2 Supplementary Figures

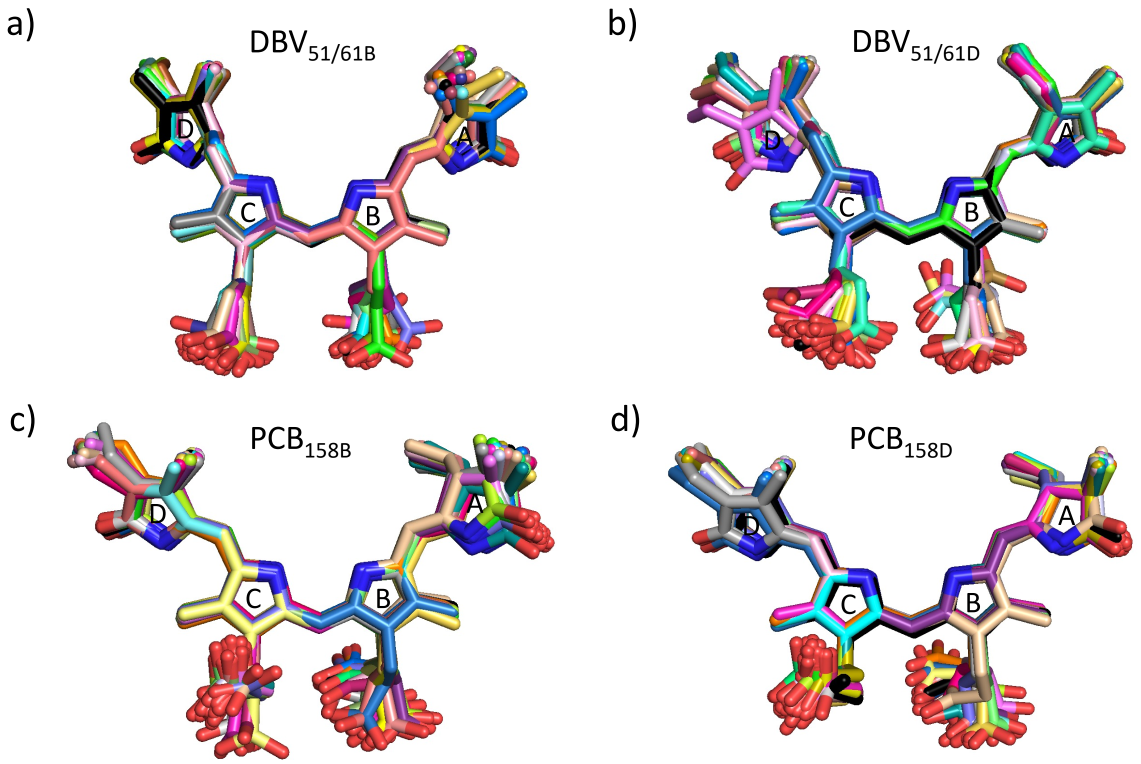

Supplementary Figure S1. Alignment of PC645 pigment structures in the crystal structure (black) and optimized along the MD simulation (colors): a) $DBV_{51/61B}$, b) $DBV_{51/61D}$, c) PCB_{158B} and d) PCB_{158D}.

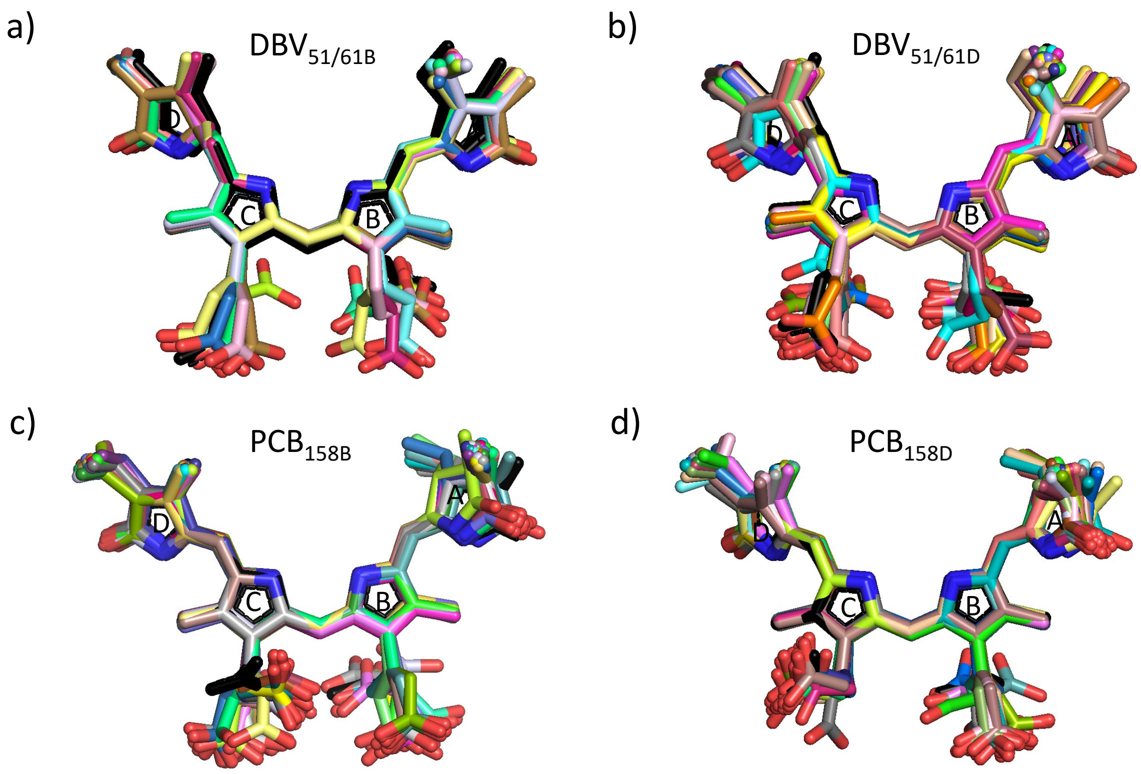

Supplementary Figure S2. Alignment of PC612 pigment structures in the crystal structure (black) and optimized along the MD simulation (colors): a) $DBV_{51/61B}$, b) $DBV_{51/61D}$, c) PCB_{158B} and d) PCB_{158D}.

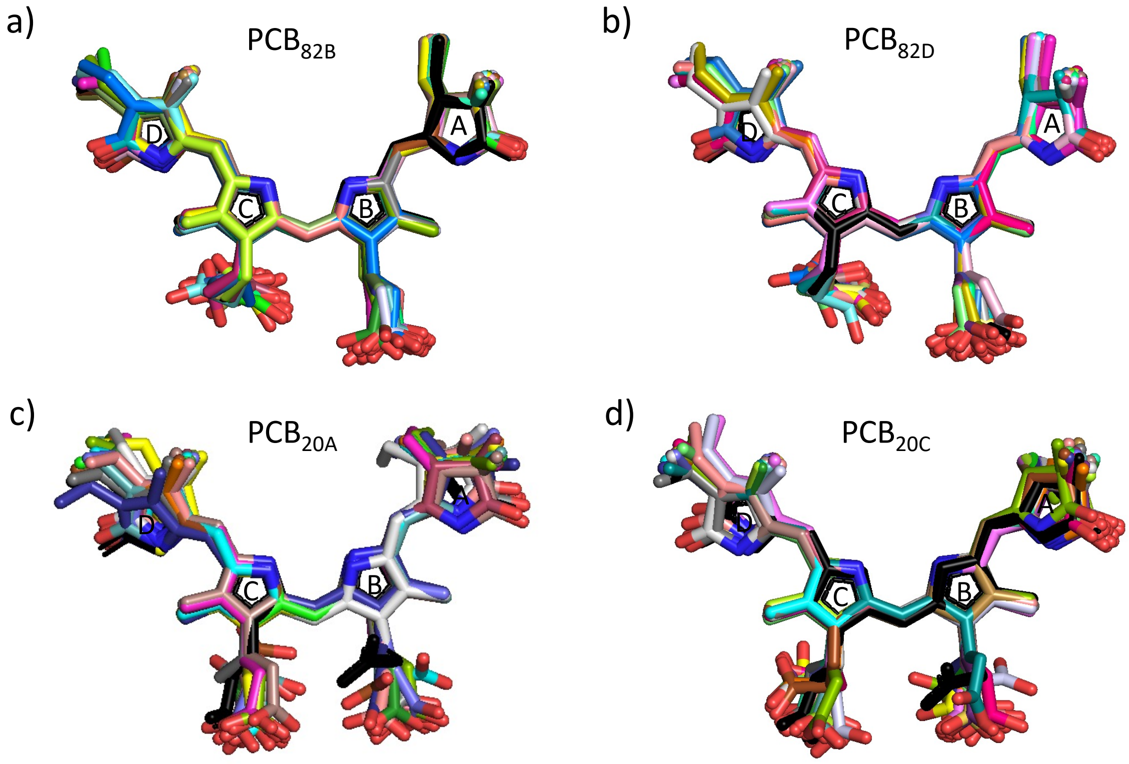

Supplementary Figure S3. Alignment of PC612 pigment structures in the crystal structure (black) and optimized along the MD simulation (colors): a) PCB_{82B}, b) PCB_{82D}, c) PCB_{20A} and d) PCB_{20C}.

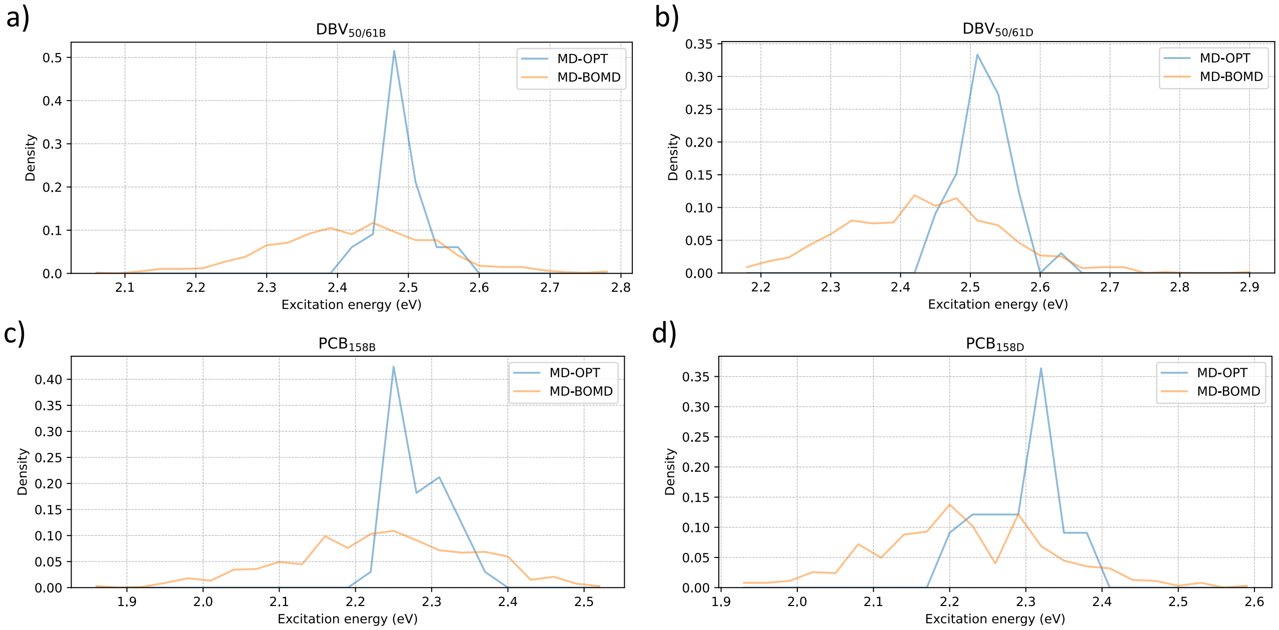

Supplementary Figure S4. Distribution of site energies for PC645 pigments computed from MD-OPT and MD-BOMD calculations: a) $DBV_{51/61B}$, b) $DBV_{51/61D}$, c) PCB_{158B} and d) PCB_{158D}.

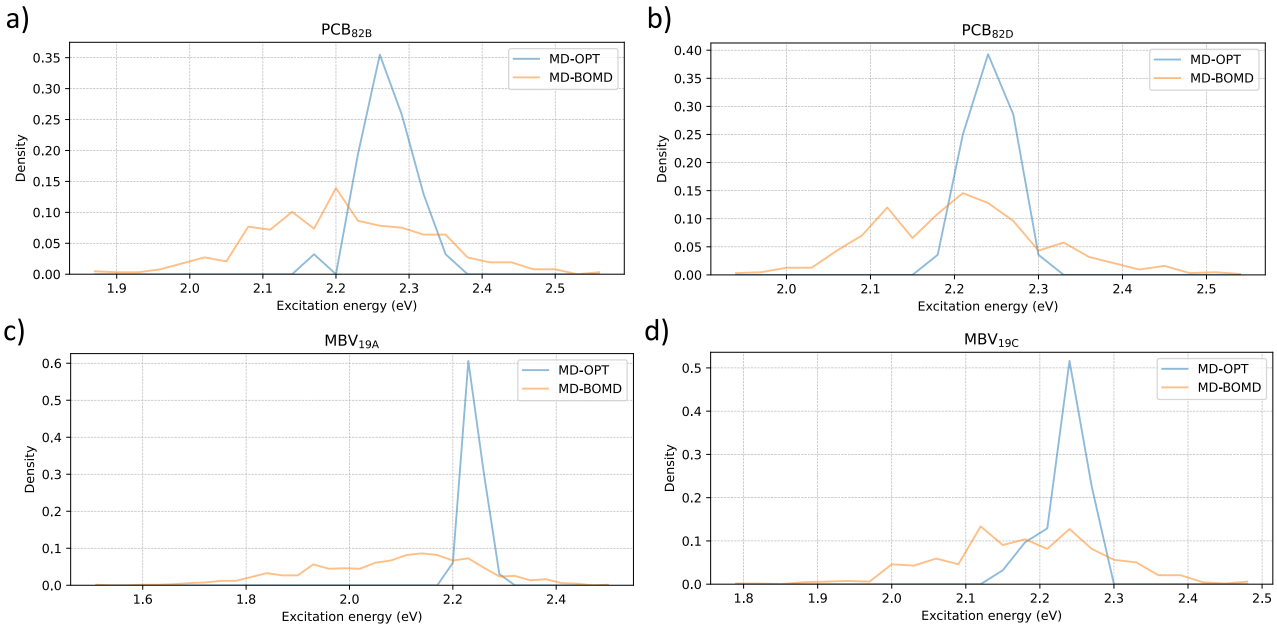

Supplementary Figure S5. Distribution of site energies for PC645 pigments computed from MD-OPT and MD-BOMD calculations: a) PCB_{82B}, b) PCB_{82D}, c) MBV_{19A} and d) MBV_{19C}.

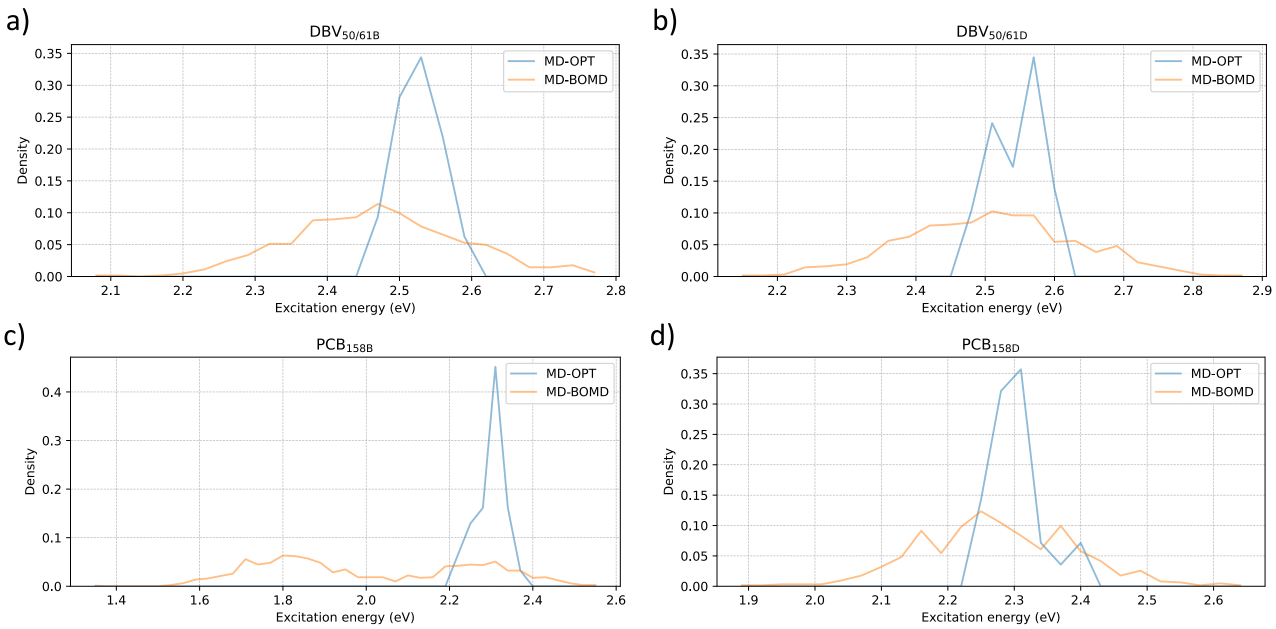

Supplementary Figure S6. Distribution of site energies for PC612 pigments computed from MD-OPT and MD-BOMD calculations: a) $DBV_{51/61B}$, b) $DBV_{51/61D}$, c) PCB_{158B} and d) PCB_{158D}.

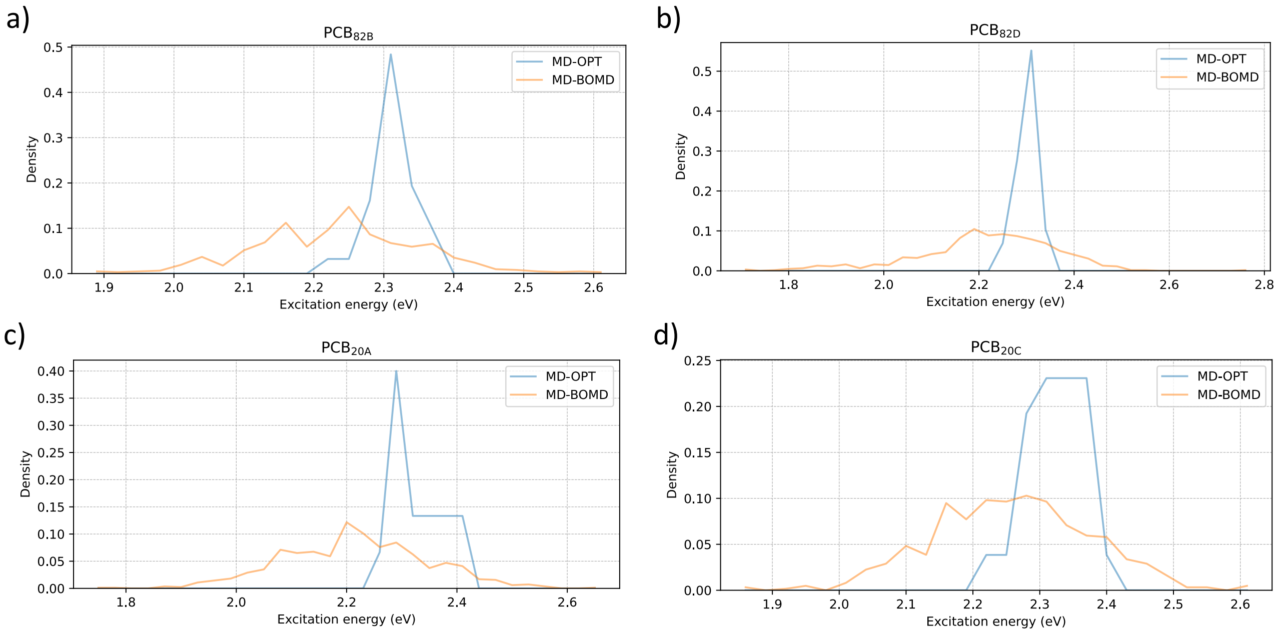

Supplementary Figure S7. Distribution of site energies for PC612 pigments computed from MD-OPT and MD-BOMD calculations: a) PCB_{82B}, b) PCB_{82D}, c) PCB_{20A} and d) PCB_{20C}.

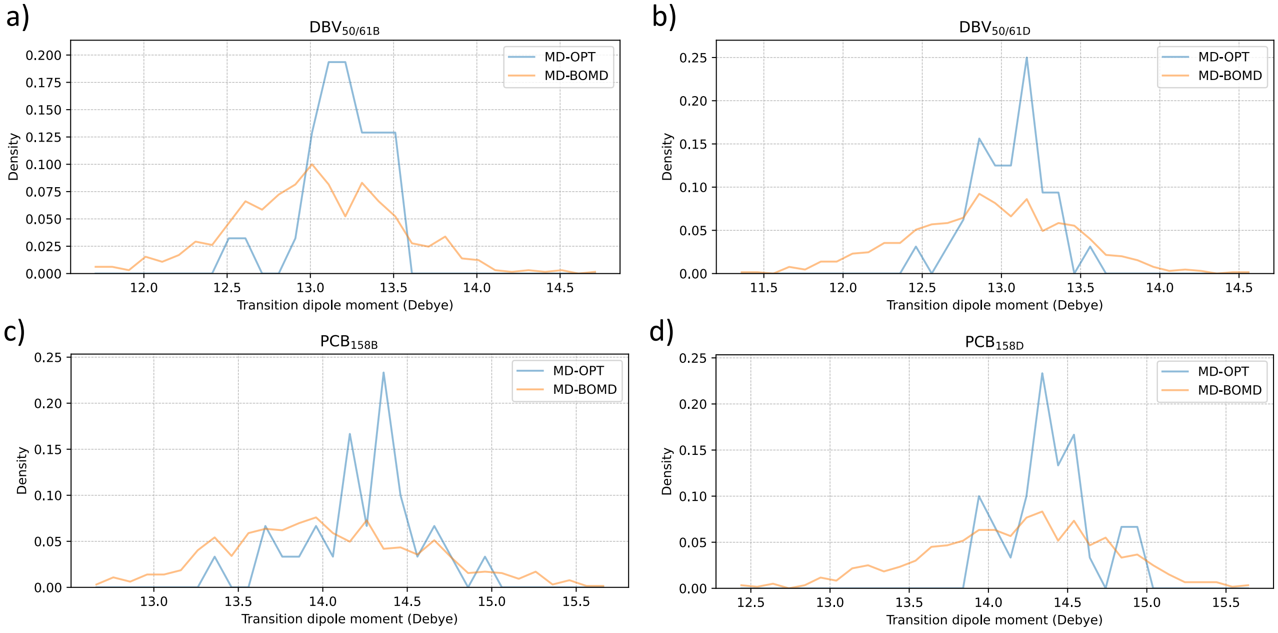

Supplementary Figure S8. Distribution of electronic transition dipole moments for PC645 pigments computed from MD-OPT and MD-BOMD calculations: a) $DBV_{51/61B}$, b) $DBV_{51/61D}$, c) PCB_{158B} and d) PCB_{158D}.

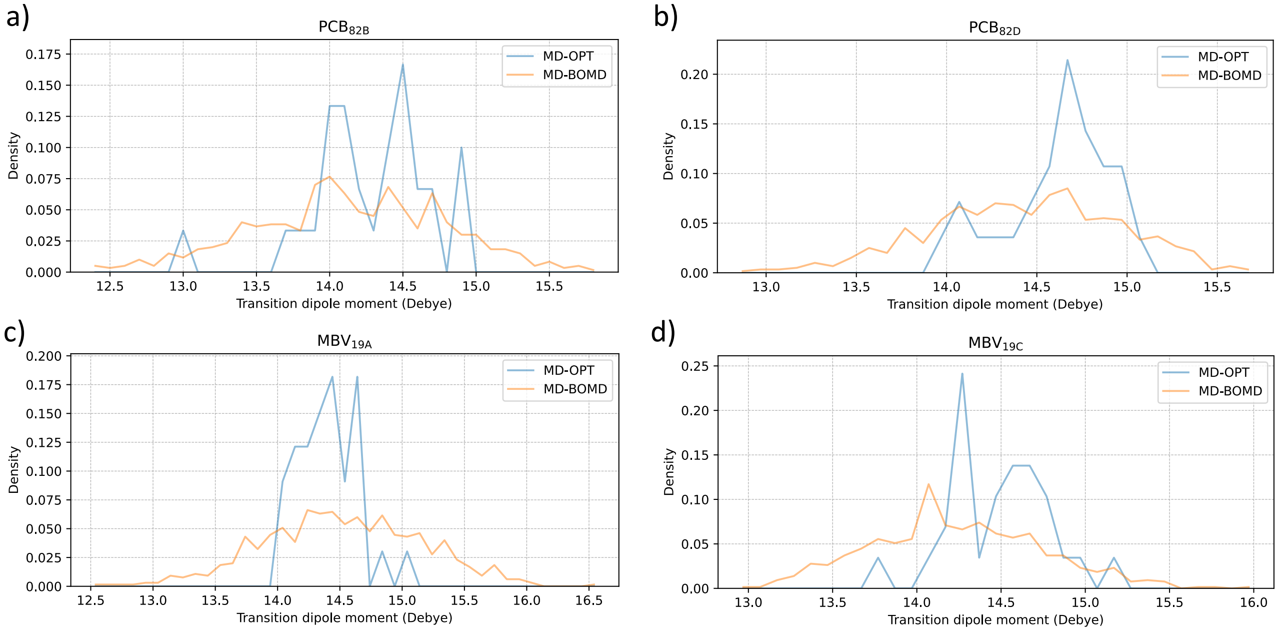

Supplementary Figure S9. Distribution of electronic transition dipole moments for PC645 pigments computed from MD-OPT and MD-BOMD calculations: a) PCB_{82B}, b) PCB_{82D}, c) MBV_{19A} and d) MBV_{19C}.

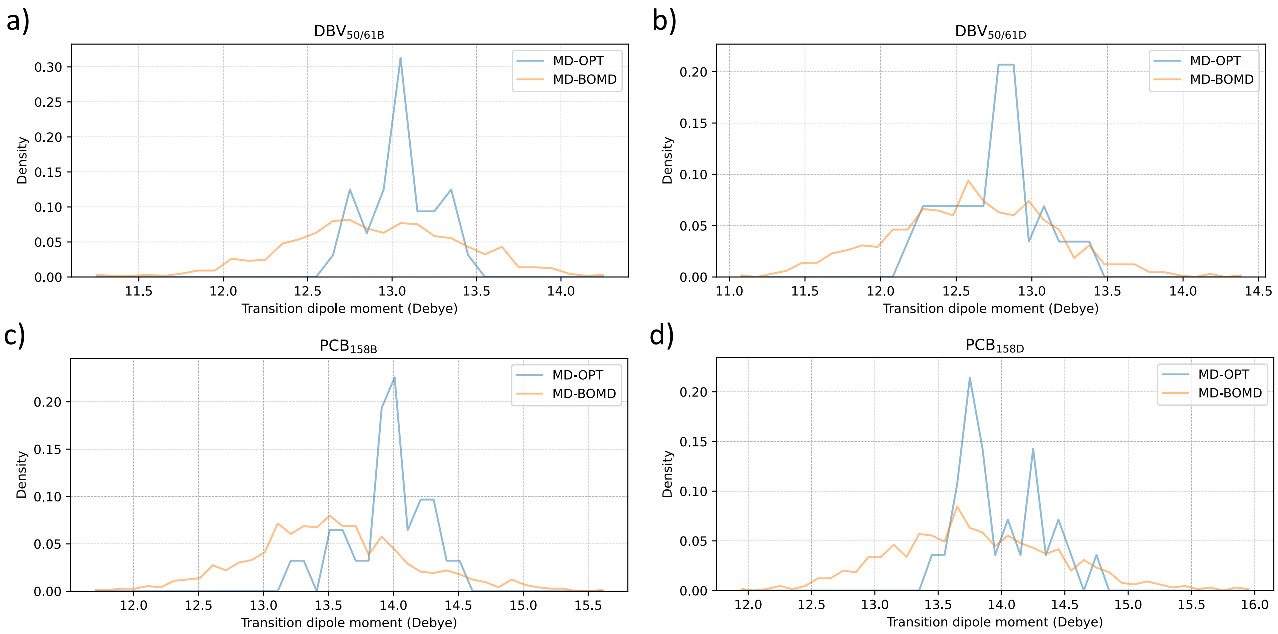

Supplementary Figure S10. Distribution of electronic transition dipole moments for PC612 pigments computed from MD-OPT and MD-BOMD calculations: a) $DBV_{51/61B}$, b) $DBV_{51/61D}$, c) PCB_{158B} and d) PCB_{158D}.

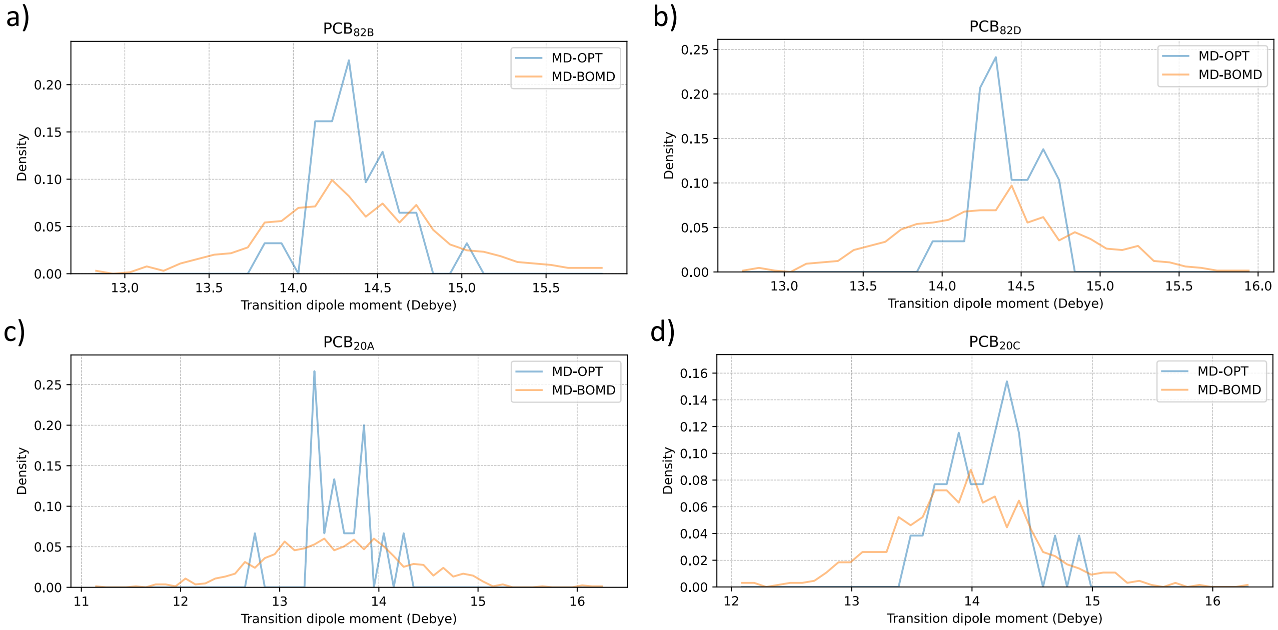

Supplementary Figure S11. Distribution of electronic transition dipole moments for PC612 pigments computed from MD-OPT and MD-BOMD calculations: a) PCB_{82B}, b) PCB_{82D}, c) PCB_{20A} and d) PCB_{20C}.

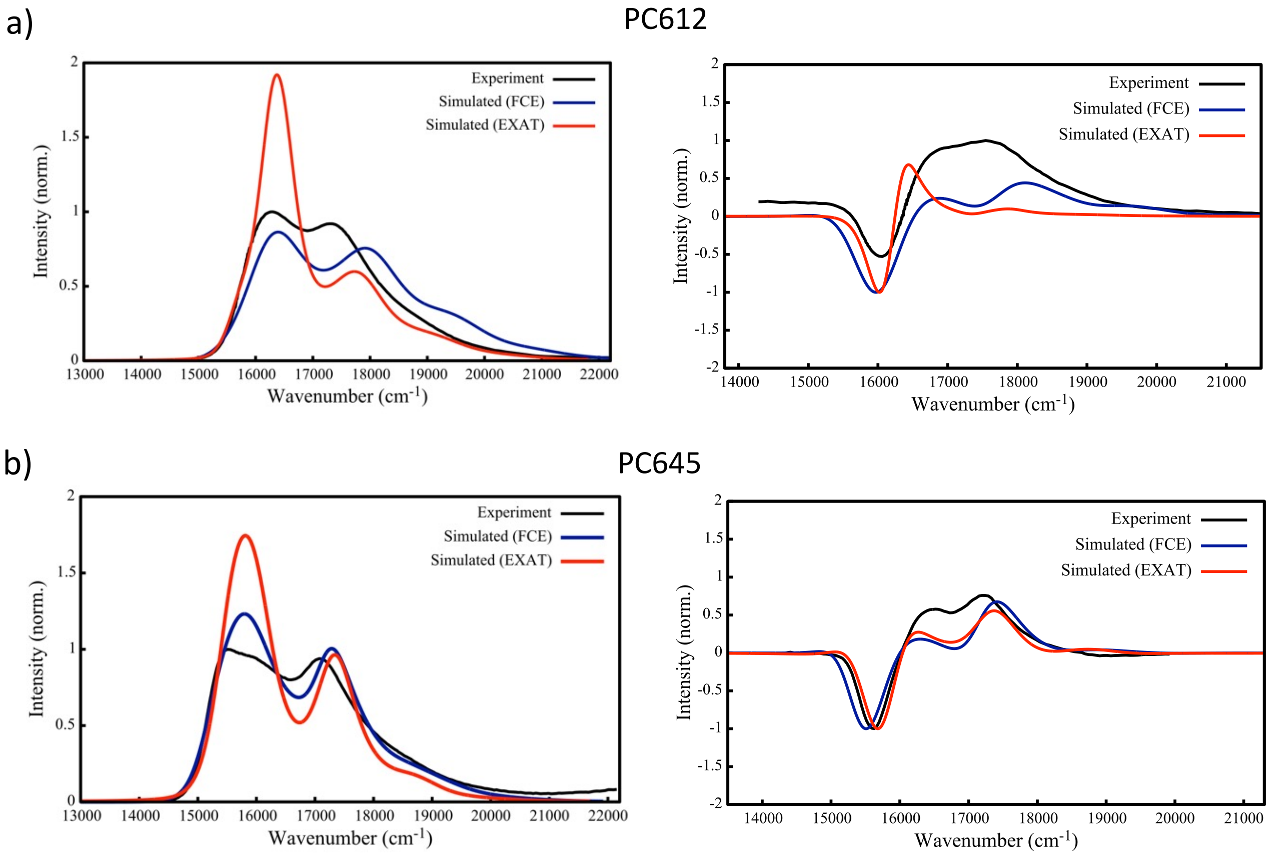

Supplementary Figure S12. Experimental[1–3] and simulated absorption (left) and circular dichroism (right) spectra of a) PC612 and b) PC645 cryptophyte antenna complexes. Simulations are based on energies and couplings computed from QM/MMPol TD-CAM-B3LYP/6-31G(d) calculations performed on MD-OPT geometries using the EXAT or FCE codes to calculate the spectra. We applied the following shifts to simulated spectra to fit the experimental bands: 2180 cm^{-1} (EXAT PC612), 1540 cm^{-1} (FCE PC612), 2300 cm^{-1} (EXAT PC645) and 2080 cm^{-1} (FCE PC645). Static disorder was modeled by applying a common $\sigma = 100$ cm^{-1}.

a) PC612

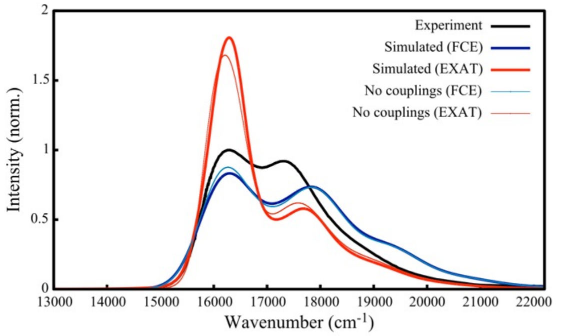

b) PC645

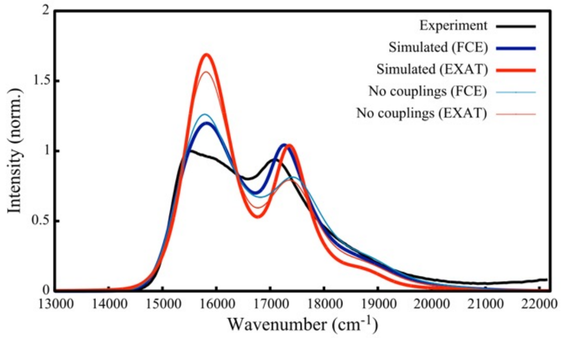

Supplementary Figure S13. Comparison of experimental[1–3] and simulated absorption spectra of a) PC612 and b) PC645 cryptophyte antenna complexes obtained including or neglecting electronic couplings between bilin pigments. Simulations are based on QM/MMPol TD-CAM-B3LYP/6-31G(d) calculations performed on MD-BOMD geometries using the EXAT or FCE codes to calculate the spectra. We applied the following shifts to simulated spectra to fit the experimental bands (couplings/no couplings values are indicated): 1600/1500 cm^{-1} (EXAT PC612), 1000/980 cm^{-1} (FCE PC612), 1700/1640 cm^{-1} (EXAT PC645) and 1480/1500 cm^{-1} (FCE PC645). Static disorder was modeled by applying a common $\sigma = 100$ cm^{-1}.

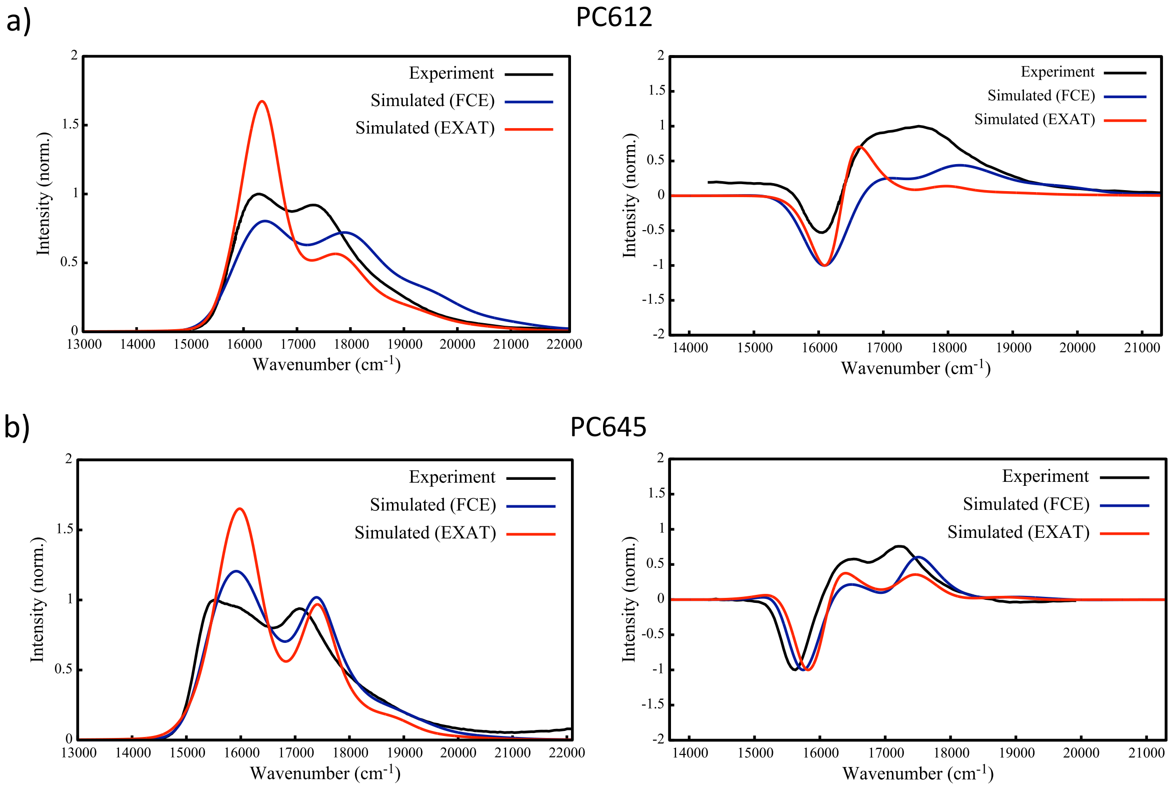

Supplementary Figure S14. Experimental[1–3] and simulated absorption (left) and circular dichroism (right) spectra of a) PC612 and b) PC645 cryptophyte antenna complexes. Simulations are based on energies and couplings computed from QM/MMPol TD-CAM-B3LYP/6-31G(d) calculations performed on MD-BOMD geometries using the EXAT or FCE codes to calculate the spectra. We applied the following shifts to simulated spectra to fit the experimental bands: 1560 cm^{-1} (EXAT PC612), 920 cm^{-1} (FCE PC612), 1620 cm^{-1} (EXAT PC645) and 1400 cm^{-1} (FCE PC645). Static disorder based on the MD-OPT σ values reported for each bilin in Table S3.

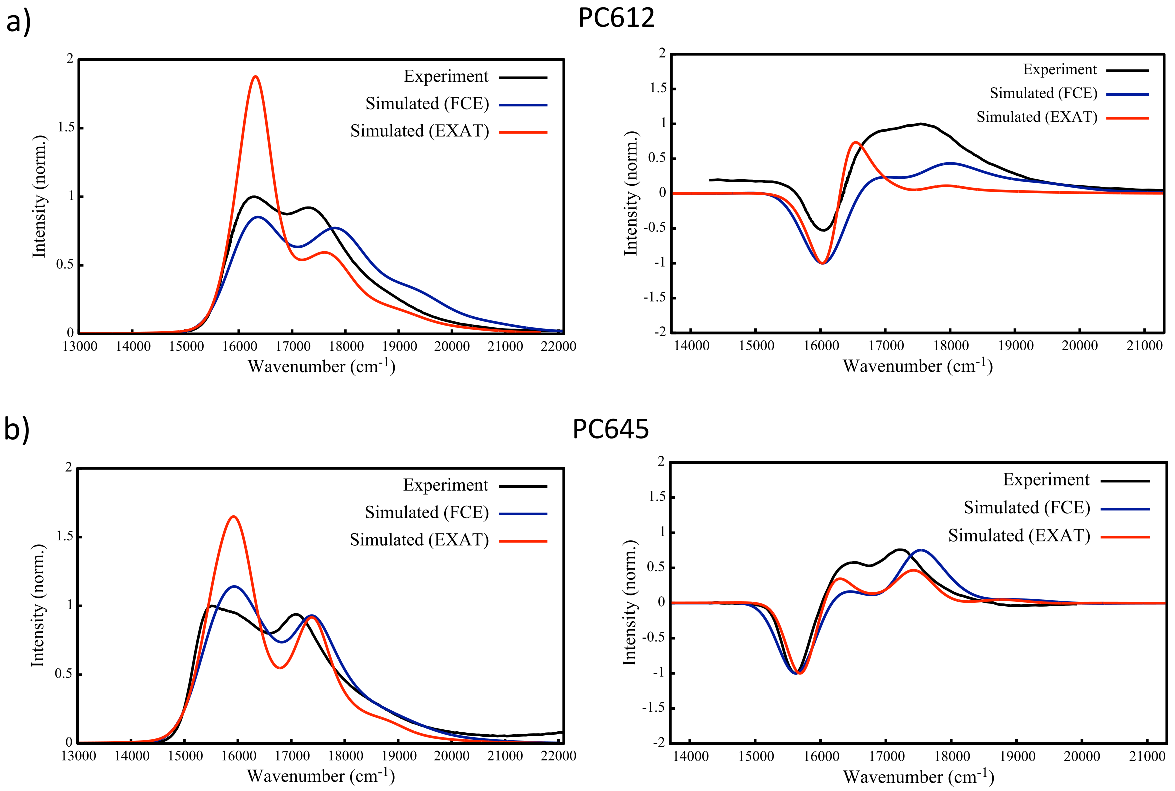

Supplementary Figure S15. Experimental[1–3] and simulated absorption (left) and circular dichroism (right) spectra of a) PC612 and b) PC645 cryptophyte antenna complexes. Simulations are based on energies and couplings computed from QM/MMPol TD-CAM-B3LYP/6-31G(d) calculations performed on MD-OPT geometries using the EXAT or FCE codes to calculate the spectra. We applied the following shifts to simulated spectra to fit the experimental bands: 2320 cm^{-1} (EXAT PC612), 1660 cm^{-1} (FCE PC612), 2260 cm^{-1} (EXAT PC645) and 1980 cm^{-1} (FCE PC645). Static disorder based on the MD-OPT σ values reported for each bilin in Table S3.

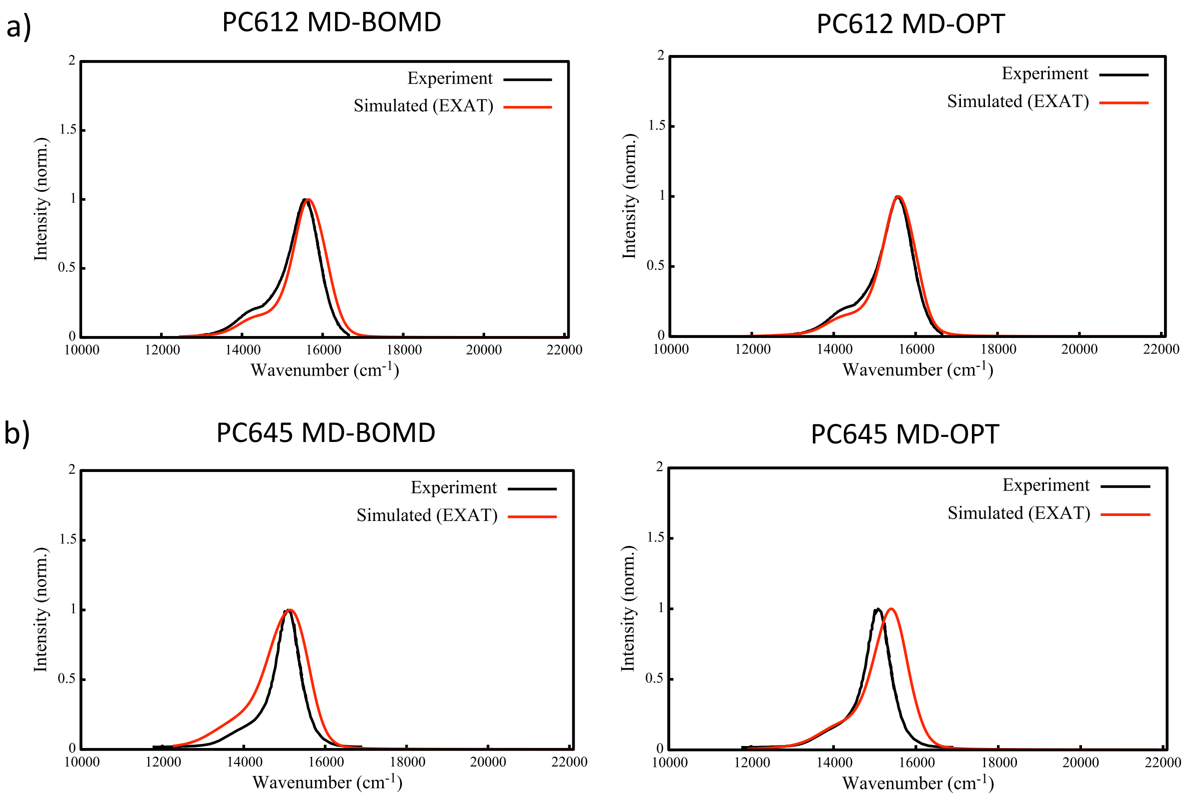

Supplementary Figure S16. Experimental[1–3] and simulated fluorescence spectra of a) PC612 and b) PC645 cryptophyte antenna complexes. Simulations are based on energies and couplings computed from QM/MMPol TD-CAM-B3LYP/6-31G(d) calculations performed on MD-BOMD (left) and MD-OPT (right) geometries using the EXAT code to calculate the spectra. We applied the following shifts to simulated spectra to fit the experimental bands: 1620 cm^{-1} (PC612 MD-BOMD), 2480 cm^{-1} (PC612 MD-OPT), 1740 cm^{-1} (PC645 MD-BOMD) and 2100 cm^{-1} (PC645 MD-OPT). Static disorder based on the MD-OPT σ values reported for each bilin in Table S3.

References

(1) Mirkovic, T.; Doust, A. B.; Kim, J.; Wilk, K. E.; Curutchet, C.; Mennucci, B.; Cammi, R.; Curmi, P. M. G.; Scholes, G. D. Ultrafast Light Harvesting Dynamics in the Cryptophyte Phycocyanin 645. *Photochemical & Photobiological Sciences* **2007**, *6* (9), 964–975. https://doi.org/10.1039/b704962e.

(2) Harrop, S. J.; Wilk, K. E.; Dinshaw, R.; Collini, E.; Mirkovic, T.; Teng, C. Y.; Oblinsky, D. G.; Green, B. R.; Hoef-Emden, K.; Hiller, R. G.; Scholes, G. D.; Curmi, P. M. G. Single-Residue Insertion Switches the Quaternary Structure and Exciton States of Cryptophyte Light-Harvesting Proteins. *Proc Natl Acad Sci U S A* **2014**, *111* (26), E2666-75. https://doi.org/10.1073/pnas.1402538111.

(3) Arpin, P.; Turner, D. B.; McClure, S. D.; Jumper, C. C.; Mirkovic, T.; Challa, J. R.; Lee, J.; Teng, C. Y.; Green, B. R.; Wilk, K. E.; Curmi, P. M. G.; Hoef-Emden, K.; McCamant, D. W.; Scholes, G. D. Spectroscopic Studies of Cryptophyte Light Harvesting Proteins: Vibrations and Coherent Oscillations. *J Phys Chem B* **2015**, *119*, 10025–10034. https://doi.org/10.1021/acs.jpcb.5b04704.
